# Illegal Hunting and Chimpanzee Distribution in the Seringbara Region of the Mount Nimba Strict Nature Reserve: A Multi‐Scale Modelling Approach

**DOI:** 10.1002/ece3.74384

**Published:** 2026-10-05

**Authors:** Lucas Degrève, Diana Erazo, Maegan Fitzgerald, Henry Didier Camara, Gnan Mamy, Simon Dellicour, Żaneta Kaszta, Kathelijne Koops

**Affiliations:** ^1^ Spatial Epidemiology Lab (SpELL) Université Libre de Bruxelles Brussels Belgium; ^2^ Division of Invertebrates, Department of Biology Royal Museum for Central Africa Tervuren Belgium; ^3^ Evolutionary Ecology Group University of Antwerp Antwerpen Belgium; ^4^ Ape Behaviour & Ecology Group, Department of Evolutionary Anthropology University of Zurich Zurich Switzerland; ^5^ Institut de Recherche sur la Biodiversité aux Monts Nimba (IReB‐MN) Lola Guinea; ^6^ Department of Microbiology, Immunology and Transplantation Rega Institute, KU Leuven Leuven Belgium; ^7^ Interuniversity Institute of Bioinformatics in Brussels Université Libre de Bruxelles, Vrije Universiteit Brussel Brussels Belgium; ^8^ Department of Biological Sciences Northern Arizona University Flagstaff Arizona USA

**Keywords:** boosted regression trees, chimpanzee, conservation, ecological niche modelling, generalised linear models, Guinea, multi‐scale niche models

## Abstract

Guinea is home to approximately two‐thirds of the remaining Critically Endangered western chimpanzee population, and the Mount Nimba Strict Nature Reserve has been identified as one of eight West African regions with exceptionally high chimpanzee densities. Despite its protected status, the reserve faces three major threats: subsistence agriculture, poaching, and industrial mining. This study focuses on understanding chimpanzee habitat use in relation to human hunting activities within the Seringbara region of the reserve. Using GPS data on chimpanzee feeding/travelling, nesting, and human hunting evidence, we develop multi‐scale ecological niche models for each activity type using both generalised linear models and a boosted regression tree approach. The models identified potential illegal hunting areas, zones of high chimpanzee activity, and the spatial overlap between them. Chimpanzee activities were primarily predicted at altitudes above 750 m, in areas with a high Enhanced Vegetation Index and steeper slopes, while hunting activities were less prevalent in areas with dense tree cover. Both chimpanzees and hunters avoid research camps, but chimpanzees show a stronger avoidance of human presence overall. High elevations, while offering relative refuge from human disturbance, also emerge as zones of greatest predicted chimpanzee‐hunter encounter probability. Our findings offer key insights for conservation planning, identifying potential zones for targeted conservation and monitoring efforts. Specifically, areas with significant overlap of chimpanzee and human hunting activities qualify as strategic areas for concerted conservation efforts.

## Graphical abstract

We modelled multi‐scale habitat use for chimpanzee feeding/travelling, nesting, and human hunting in the Seringbara region of Mount Nimba (Guinea) using GPS data with GLMs and boosted regression trees. Chimpanzee activity concentrated above 750 m on steeper slopes with high EVI, hunting was less prevalent in dense tree cover, both avoided research camps, and predicted encounter risk peaked at higher elevations. The resulting maps identify likely illegal hunting areas, chimpanzee core areas, and their overlap‐pinpointing spatial priorities for targeted monitoring and protection.

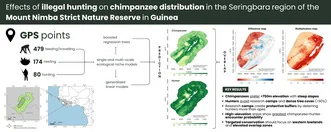

## 1 Introduction

The western chimpanzee ( *Pan troglodytes verus* ; Schwarz, 1934) is the only one of four chimpanzee subspecies listed as ‘Critically Endangered’ on the International Union for Conservation of Nature (IUCN) Red List of Threatened Species (Humle et al. 2016). Between 1990 and 2014, the population of this subspecies experienced an alarming 80% decline (Kühl et al. 2017; Heinicke, Mundry, Boesch, Amarasekaran, et al. 2019), resulting in an estimated population of 52,800 individuals (Heinicke, Mundry, Boesch, Amarasekaran, et al. 2019). Additionally, the western subspecies has already disappeared from three of the eleven countries it historically inhabited (Ginn et al. 2013; Campbell and Houngbedji 2015) and its range shrunk by 20% over 24 years (Kühl et al. 2017). This decline threatens western chimpanzees with widespread extirpation and significant loss of genetic and behavioural diversity (IUCN SSC Primate Specialist Group 2020; Kühl et al. 2019), a trend likely to continue given that over 10% of the population inhabits areas designated for large‐scale infrastructure projects (Heinicke, Mundry, Boesch, Amarasekaran, et al. 2019).

The primary threats to western chimpanzees include subsistence agriculture, poaching, and industrial mining (IUCN SSC Primate Specialist Group 2020; Fitzgerald et al. 2018). Guinea hosts the largest population with around 33,140 individuals, representing nearly two‐thirds of the global western chimpanzee population (Heinicke, Mundry, Boesch, Amarasekaran, et al. 2019). The Mount Nimba Strict Nature Reserve (MNSNR), spanning Côte d'Ivoire and Guinea, has been identified as one of eight regions in western Africa with exceptionally high chimpanzee densities (Heinicke, Mundry, Boesch, Hockings, et al. 2019; Kühl et al. 2017; IUCN SSC Primate Specialist Group 2020). Despite its protected status permitting only scientific access, the MNSNR in Guinea faces all three of these primary threats to western chimpanzees (Koops et al. 2023; World Heritage Committee 2023; Matsuzawa et al. 2011).

Although laws and local customs strictly prohibit chimpanzee hunting (Sugiyama 1978; Kortlandt 1986), hunters from outside the region who may not share local taboos pose a significant threat. While hunting in the area primarily targets smaller mammals rather than chimpanzees (Matsuzawa et al. 2011), poaching activities can still disturb and threaten chimpanzees through by‐catch, injuries, and human disturbance (IUCN SSC Primate Specialist Group 2020). Even where chimpanzee‐targeted hunting is minimal, these apes may exhibit fine‐scale spatiotemporal adjustments to human activities (Bryson‐Morrison et al. 2017). Previous studies have indicated that in regions where hunting has occurred or is ongoing, chimpanzees tend to spatially avoid human presence (Matthews and Matthews 2004). This underscores the potential influence of human disturbance on chimpanzee distribution and behaviour (Kühl et al. 2019).

Here, we investigated the effect of human hunting activities on chimpanzees ranging in the Seringbara study area on the western flank of the Nimba Mountain range, which covers around 30 km^2^ within the MNSNR (Fitzgerald et al. 2018). This long‐term chimpanzee study site was established in 2003 (Koops 2011). Our study specifically aimed to investigate (i) the spatial and biophysical factors influencing human hunting activity, (ii) the spatial and biophysical factors influencing chimpanzee nesting and feeding/travelling patterns, and (iii) the impact of hunting on chimpanzee distribution within the Seringbara region of the MNSNR.

To achieve these objectives, we analysed Global Positioning System (GPS)‐based occurrences of human and chimpanzee presence data collected over twelve months (August 2022–August 2023), alongside various fine‐scale geospatial layers. Our dataset comprises locations of direct and indirect evidence of chimpanzee and human occurrences split into three categories reflecting distinct uses of the reserve: ‘chimpanzee feeding and travelling’, ‘chimpanzee nesting’, and ‘human hunting’. For each category, we conducted ecological niche modelling (ENM) analyses to estimate local ecological suitability for chimpanzee habitat use and probability of occurrence for human hunting. Specifically, we performed and compared two modelling approaches: generalised linear models (GLM), known for their interpretability and statistical robustness, and boosted regression trees (BRT), a machine learning approach capable of capturing complex, non‐linear relationships (Guisan et al. 2002; Elith et al. 2008). We used ENM to generate spatial predictions of both human and chimpanzee activities, as well as to examine potential spatial displacement of chimpanzees by human hunting activities. Our findings offer guidance for future conservation planning by identifying priority zones for targeted conservation and monitoring, particularly in areas where chimpanzee habitat use overlaps with human hunting activity.

## 2 Methods

### 2.1 Ethics Statement

Data collection was non‐invasive, in compliance with Guinea's laws, and received approval from the *Direction Générale de la Recherche Scientifique et l'Innovation Technologique* (DGERSIT). All research adhered to the American Society of Primatologists (ASP) principles for the ethical treatment of nonhuman primates.

### 2.2 Study Area

The MNSNR is a UNESCO World Heritage Site in Danger (World Heritage Committee 2023). It encompasses most of the Nimba Mountain range in Guinea and parts of Côte d'Ivoire on the southeastern side of the mountain range. The reserve spans approximately 175 km^2^, comprising diverse terrain like evergreen forests, rocky peaks, rugged cliffs, granite outcrops, steep river valleys, high‐altitude savannahs, and rolling hilltops (Guillaumet and Adjanohoun 1971; Koops 2011; Kormos et al. 2003; Fitzgerald et al. 2018). It hosts unique flora and fauna, including the Critically Endangered Mount Nimba viviparous toad, *Nimbaphrynoides occidentalis* (Angel 1943), and western chimpanzee (World Heritage Committee 2023).

The Seringbara study area (7.634° N, 8.425° W) is located on the western side of the Nimba Mountains within the MNSNR (Figure 1; Fitzgerald et al. 2018). It comprises primary tropical forest, transitioning to terrestrial herbaceous vegetation, montane forest, and high‐altitude grasslands on steeper terrain (Koops 2011). Elevation varies from 595 to 1752 m, with a climate characterised by a rainy season from February to October and a 3‐month dry season from November to January (Koops, McGrew, de Vries, and Matsuzawa 2012; Koops, McGrew, Matsuzawa, and Knapp 2012). The Seringbara region harbours two chimpanzee communities (i.e., Gahtoy, Tongbongbon) of approximately 89 individuals (Koops et al. 2023). Chimpanzee research has been ongoing since 2003, but due to extremely mountainous terrain, the chimpanzees remain largely unhabituated to human observers (Koops 2011).

**FIGURE 1:**
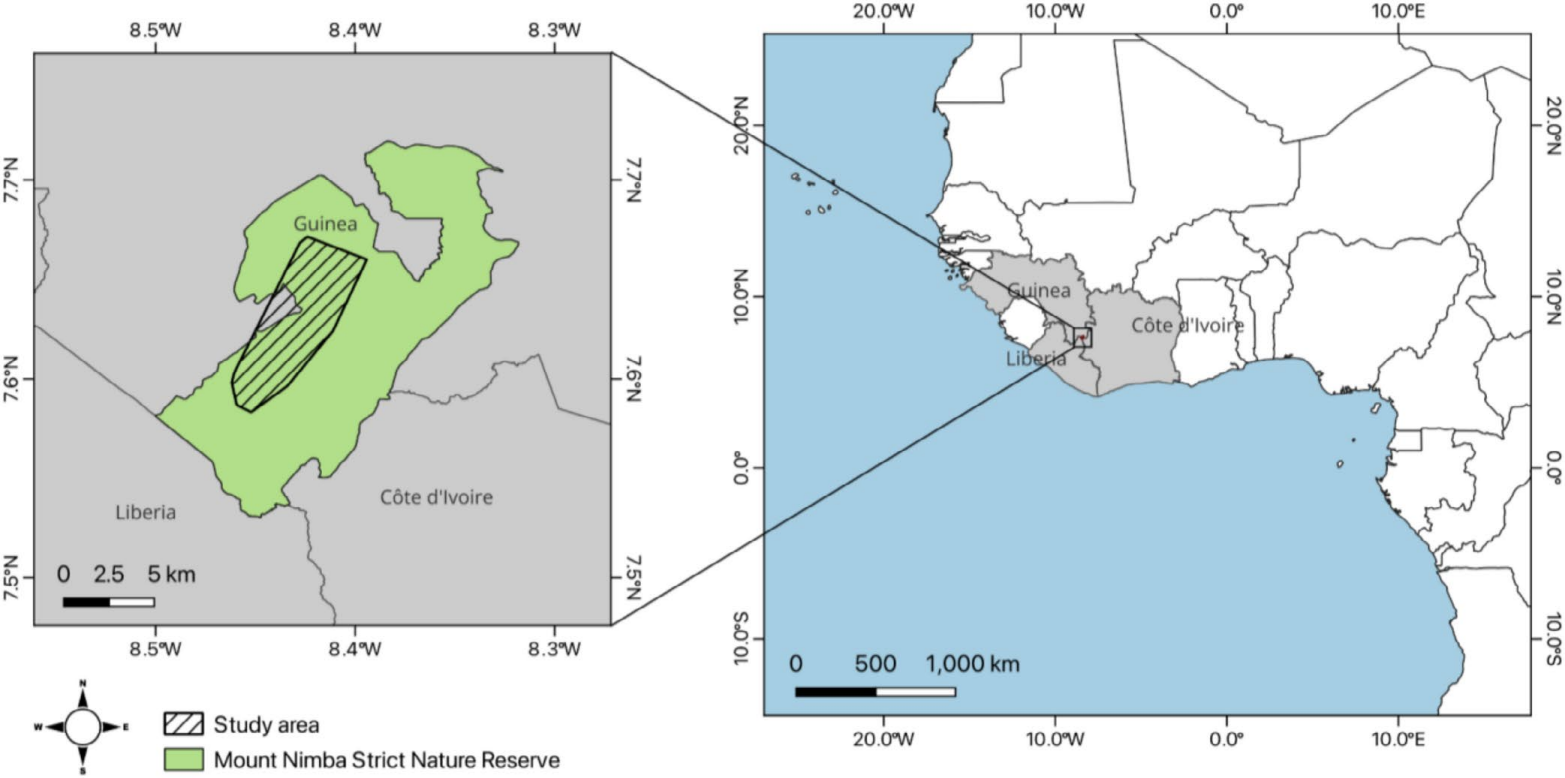
Location of the Seringbara study area within the Guinean part of the Mount Nimba Strict Nature Reserve (MNSNR) in West Africa (Fitzgerald et al. 2018).

### 2.3 Data Collection

Data were collected over twelve months (August 2022–August 2023). Direct and indirect evidence of chimpanzee and human presence were documented using handheld GPS devices. Chimpanzee evidence (max. 2 months old) included nests, fresh faeces, knuckle prints, resting areas, trails, feeding traces, and direct observations. Hunting evidence included empty bullet casings, bullet marks, snare traps, hunting camps, hunter tracks, hunting campfires, bushfires, and direct observations. Multiple teams conducted daily chimpanzee searches (20 days/month), with each team covering a section of the study area; complete spatial coverage was achieved cumulatively over the course of each monthly survey cycle rather than on any single day. A total of 901 occurrence points were recorded: 633 feeding/travelling points, 185 nesting points (day and night nests), and 83 hunting points. We distinguished between nesting and feeding/travelling activities as they are likely affected differently by human hunting activities.

### 2.4 Selection of Presence and Pseudo‐Absence Points

For presence points, we retained one location per 30 m pixel, reducing feeding/travelling points to 479, nesting points to 174, and hunting points to 80. Barbet‐Massin et al. (2012) recommended using the same number of pseudo‐absences as presences for classification techniques such as BRT. Although GLM performs slightly better with a larger set of pseudo‐absences, we opted to use the same number of pseudo‐absence and presence points for each modelling approach to facilitate comparison. For each activity type we ran 100 iterations of models, each time using a different set of pseudo‐absences.

Pseudo‐absence points were generated using GPS tracks surveyed by local Guinean researchers (August 2022–August 2023), randomly sampled only in raster cells crossed by these tracks and weighted according to their frequency (Figure 2). This approach avoided sampling in non‐screened areas where absence could simply indicate lack of screening. Tracks were cleaned in QGIS 3.30.0 to remove duplicates. Pseudo‐absence points within a 30 m wide buffer of a presence point were discarded. This buffer distance aligns with definitions used in chimpanzee research (Koops, McGrew, de Vries, and Matsuzawa 2012; Furuichi et al. 2001), specifically the categorisation of a nest group, defined as nests of similar age located no more than 30 m apart (Humle 2003). Additionally, for consistency across models and in line with our raster resolution of 30 m, we used the same distance for both hunting and feeding/travelling models.

**FIGURE 2:**
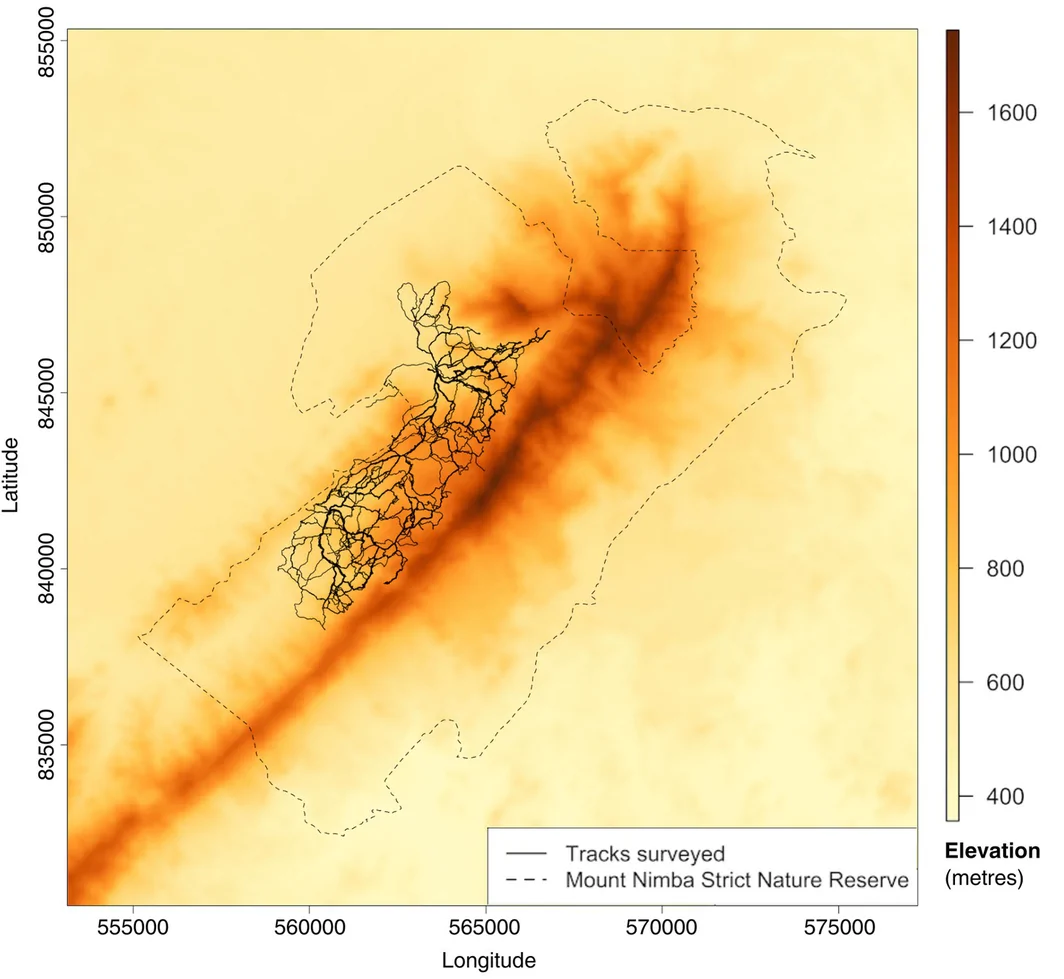
Location of the one‐year GPS tracks surveyed within the Mount Nimba Strict Nature Reserve, overlaid on the elevation raster layer measured in metres.

### 2.5 Environmental Data & Multi‐Scale Approach

We selected 16 variables potentially influencing both chimpanzee and human activities in the Nimba Mountain range (Fitzgerald et al. 2018; Table S1). Most predictors function as proxies for underlying gradients of human accessibility and microhabitat quality rather than as direct causal drivers of chimpanzee habitat selection, owing to the rarity or absence of direct causal open‐source fine‐scale layers. The predictor set therefore reflects the practical limits of currently available data rather than a conceptual preference for indirect measures. Predictor selection followed Fitzgerald et al. (2018) and was guided by established knowledge of chimpanzee ecology and human hunting behaviour in the Nimba Mountains. Topographic variables (slope, elevation, TPI, CTI, aspect, CHILI) were included because terrain steepness and position strongly influence both chimpanzee habitat use and hunter movement. For chimpanzees, steep slopes are generally associated with reduced human accessibility and are preferred for nesting and foraging (Koops, McGrew, de Vries, and Matsuzawa 2012; Fotang et al. 2021). Vegetation variables (EVI, tree cover, wetness) were included as proxies for primary forest cover and food resource availability, both key determinants of chimpanzee distribution (Fitzgerald et al. 2018; Koops 2011). Distance‐based variables (distance to village, road, river, and research camp) were included as direct or proxy measures of human accessibility and disturbance intensity, expected to negatively influence chimpanzee presence and shape hunter access depending on terrain and infrastructure (Campbell et al. 2011). Rasters representing each variable were obtained and resampled to 30 m resolution using Google Earth Engine (Gorelick et al. 2017). Euclidean distance rasters (distance to village, road, river, and research camp) were generated with QGIS using vector data from various providers. All variables were (re)projected to WGS 84/UTM zone 29N (EPSG:32629).

Recognising that species‐habitat relationships are scale‐dependent (Levin 1992; Wiens 1976, 1989), we implemented a multi‐scale (MS) approach that has been shown to enhance predictive power compared to single‐scale (SS) models (Timm et al. 2016; Wan et al. 2017; Mateo Sanchez et al. 2014). Despite its importance, less than 5% of habitat modelling studies employ robust methods to optimise scale in multivariate relationships (McGarigal et al. 2016). Scalar relationships are fundamental for understanding habitat use and invaluable for conservation management and reserve design (Macdonald et al. 2019; Ash et al. 2021).

Thus, we transformed each variable—excluding distance variables—into seven MS covariates: 30 m, 100 m, 250 m, 500 m, 1 km, 1.5 km, and 2 km using a Gaussian kernel neighbourhood applied to each raster cell with the ‘ee.Kernel.gaussian’ function in Google Earth Engine. SS models use each predictor at its native 30 m resolution, whereas MS models replace each predictor with its scale‐optimised version. To identify the optimal spatial scale for each environmental predictor, we fitted univariate GLM and BRT models in R (version 4.4.1; R Core Team 2024) independently for each of the three response variables (chimpanzee feeding/travelling, nesting, and human hunting), producing six independent sets of scale‐optimised predictors. Within each combination, we retained the scale that maximised the Area Under the Receiver Operating Characteristic (ROC) Curve (AUC; Tables S2–S4).

To assess multicollinearity of the scale‐optimised variables, we computed Spearman's correlation coefficients for each model and then averaged these coefficients across all 100 model iterations by variable and modelling approach. Machine learning methods such as BRT usually present the advantage to efficiently handle interaction effects between predictive variables (Elith et al. 2008). Nevertheless, to enhance the interpretability of the results, variables exhibiting near‐identical correlations (|*r*| > 0.9) were excluded from the BRT analyses. For GLM, when two variables showed high correlation (|*r*| > 0.8), only one was retained (Figure S8), selected based on interpretability and principal component analysis (PCA).

### 2.6 Ecological Niche Modelling

ENM approaches were employed to serve two complementary purposes: (i) an inferential goal, identifying the spatial and biophysical factors influencing chimpanzee feeding/travelling, nesting, and human hunting activity; and (ii) a predictive goal, generating spatial suitability maps to assess the degree of overlap between hunting and chimpanzee distribution across the study area. Two complementary modelling approaches, GLM and BRT, were used to serve these goals and to evaluate the robustness of results across methods. ENM links species occurrence data with environmental variables to generate spatial predictions of ecological suitability, which ranges from 0 to 1 (Elith and Leathwick 2009; Guisan and Thuiller 2005). Various statistical approaches are available for constructing ENM (Guisan et al. 2017; Valavi et al. 2022). While regression methods were initially used in ENM (Guisan et al. 2002), machine learning tools have gained prominence (Stupariu et al. 2022; Chiaverini et al. 2023) for their numerous advantages (Elith et al. 2008). In this study, both GLM and BRT were used for conducting ENM. GLM extends linear models to account for non‐linearity and non‐constant data variance (Guisan et al. 2002), making them suitable for modelling species‐habitat relationships, which often deviate from normal distributions (Austin 1987). BRT captures complex non‐linear relationships across various predictor types while considering variable interactions (Elith et al. 2008), making it a robust approach (Elith et al. 2006).

For GLM analyses, we used the ‘dredge’ function in the ‘MuMIn’ R package to automate model selection, generating multiple submodels and evaluating all possible predictor variable combinations. Models with a delta corrected Akaike Information Criterion (AICc) lower than 2 were retained and averaged using the ‘model.avg’ function (MuMIn version 1.48.4; Barton 2024). We conducted 100 runs, each with a different set of pseudo‐absences, using 85% of locations for training and 15% for validation. GLM predictive performance was evaluated using AUC and coefficient of determination (*R* ^2^). Predictor importance was assessed using model‐averaged coefficients and 95% confidence intervals (CI), computed using the full average method (Burnham and Anderson 2002). Relative variable importance was quantified as the sum of AICc weights across all models containing each predictor (‘sw’ function, MuMIn; Barton 2024). Predictors whose 95% CI did not overlap zero were considered to show a clear directional association with the response variable. We assessed overdispersion by calculating the ratio of residual deviance to residual degrees of freedom, which ideally should be less than 1, but at most less than 2. AUC values were computed using the ‘roc’ function (pROC package version 1.18.5; Robin et al. 2023).

BRT analyses were implemented using the ‘gbm.step’ function of the ‘dismo’ package version 1.3‐14 (Hijmans et al. 2023). To mitigate spatial autocorrelation and prevent overfitting, we implemented a spatial cross‐validation using the ‘blockCV’ package (version 3.1‐4; Valavi et al. 2018), which partitions the binary presence/pseudo‐absence data into spatial blocks with different folds for each BRT run. With spatial block cross‐validation, prior removal of covariates based on pairwise correlation or correction for spatial autocorrelation is not compulsory, as the procedure itself accounts for the spatial structure in the data (Valavi et al. 2018). To confirm that this procedure successfully removed residual spatial autocorrelation, correlograms were computed on BRT residuals for all six models using the ‘ncf’ package version 1.3‐3 (Bjørnstad 2023). Moran's I values were at or below zero across all distance classes (0–10 km) for all models, confirming the absence of positive residual spatial autocorrelation. One hundred independent replicate BRT models were trained. BRT predictive performance was evaluated using AUC, *R* ^2^, and the prevalence‐pseudoabsence‐calibrated Sørensen's index (SI_ppc_; Sørensen 1948). AUC values were computed directly using the ‘gbm.step’ function. Since AUC can be sensitive to species prevalence potentially biasing results (Lobo et al. 2008; Jiménez‐Valverde 2012; Jiménez‐Valverde 2014), we also computed SI_ppc_, which is less affected by prevalence variations. AUC were computed for both training and test datasets across both modelling approaches.

To generate predictions, we used the ‘predict’ function of the ‘stats’ package (version 4.2.2) for GLM and the ‘predict.gbm’ function of the ‘gbm’ package (version 2.1.9; Ridgeway 2024) for BRT, averaging across 100 simulation runs. To address sampling bias, areas above the highest surveyed elevation were masked out. For GLM, which produced unrealistically high predictions outside the reserve, areas outside the minimum convex polygon (MCP) surrounding tracks (expanded by a 2 km buffer) were also masked. For BRT, with better extrapolation capabilities, only elevations and areas outside a 2 km buffer of the MNSNR boundary were masked. This buffer distance was chosen to match our largest MS covariate (2 km), ensuring that the scale‐dependent species‐habitat relationships were appropriately represented in the predicted ecological suitability maps.

Finally, we intersected spatial predictions to examine potential displacements and overlap between hunting activities and both types of chimpanzee activities. This involved: (1) subtracting chimpanzee models surfaces from hunting model surfaces to highlight niche differences, and (2) multiplying surfaces to identify overlap areas. We calculated the averaged absolute difference and correlation coefficient between predictive surfaces, with correlations over 0.6 considered indicative of strong spatial association. These analyses were conducted within each modelling approach (i.e., GLM and BRT) for both SS and MS models.

## 3 Results

### 3.1 Model Performances

We trained six BRT models and six binomial GLMs, which resulted in a total of 12 different types of models. The cv AUC values highlighted a lower predictive performance for the BRT models compared to the GLM (Table 1). However, BRT models showed higher mean AUC values for training datasets and superior *R* ^2^ values (Table 1). Scale‐optimised BRT models consistently outperformed SS counterparts based on the comparison of cv AUC metrics (‘cv AUC’ reported in Table 1). In contrast, for the GLM analyses, enhanced predictive performance using a MS approach was observed only for the hunting models. The other two chimpanzee MS GLMs displayed comparable, though slightly lower, predictive performance than those obtained using the SS approach (Table 1). BRT predictions demonstrated greater extrapolation capacity, allowing maps to be generated for the entire MNSNR with a 2 km buffer (Figure 4; Figures S1b–S6b), while GLM prediction maps were confined to a minimum convex polygon around surveyed tracks with a 2 km buffer (Figure 3; Figures S9–S14).

**TABLE 1:** Predictive performance of the models trained using both BRT and GLM approaches.^a^

Model name	AUC	cv AUC	*R* ^2^ (training)	*R* ^2^ (test)	SI_ppc_
BRT_feed_30m	0.833 (0.010)	0.667 (0.022)	0.261 (0.025)	—	0.770 (0.010)
BRT_feed_scale	0.848 (0.011)	0.695 (0.022)	0.298 (0.028)	—	0.788 (0.009)
BRT_nest_30m	0.926 (0.016)	0.706 (0.042)	0.420 (0.076)	—	0.861 (0.020)
BRT_nest_scale	0.922 (0.018)	0.717 (0.043)	0.442 (0.086)	—	0.859 (0.019)
BRT_hunt_30m	0.956 (0.016)	0.696 (0.059)	0.475 (0.115)	—	0.892 (0.025)
BRT_hunt_scale	0.965 (0.014)	0.737 (0.058)	0.567 (0.112)	—	0.909 (0.025)
GLM_feed_30m	0.733 (0.015)	0.715 (0.043)	0.171 (0.019)	0.145 (0.057)	—
GLM_feed_scale	0.725 (0.015)	0.704 (0.045)	0.162 (0.019)	0.132 (0.058)	—
GLM_nest_30m	0.786 (0.021)	0.757 (0.070)	0.249 (0.035)	0.194 (0.114)	—
GLM_nest_scale	0.780 (0.021)	0.750 (0.065)	0.236 (0.035)	0.180 (0.103)	—
GLM_hunt_30m	0.820 (0.029)	0.753 (0.097)	0.309 (0.054)	0.179 (0.185)	—
GLM_hunt_scale	0.852 (0.028)	0.792 (0.090)	0.377 (0.059)	0.239 (0.177)	—

^a^ The table reports the area under the receiver operating characteristic curve (AUC), the cross‐validated AUC (cv AUC), the coefficient of determination (*R* ^2^) for both training datasets (BRT and GLM) and test datasets (GLM only), as well as the prevalence‐pseudoabsence‐calibrated Sørensen.s index (SIppc; BRT only). All metrics are averaged across 100 replicate models. Standard deviations for each metric are provided in parentheses.

**FIGURE 3:**
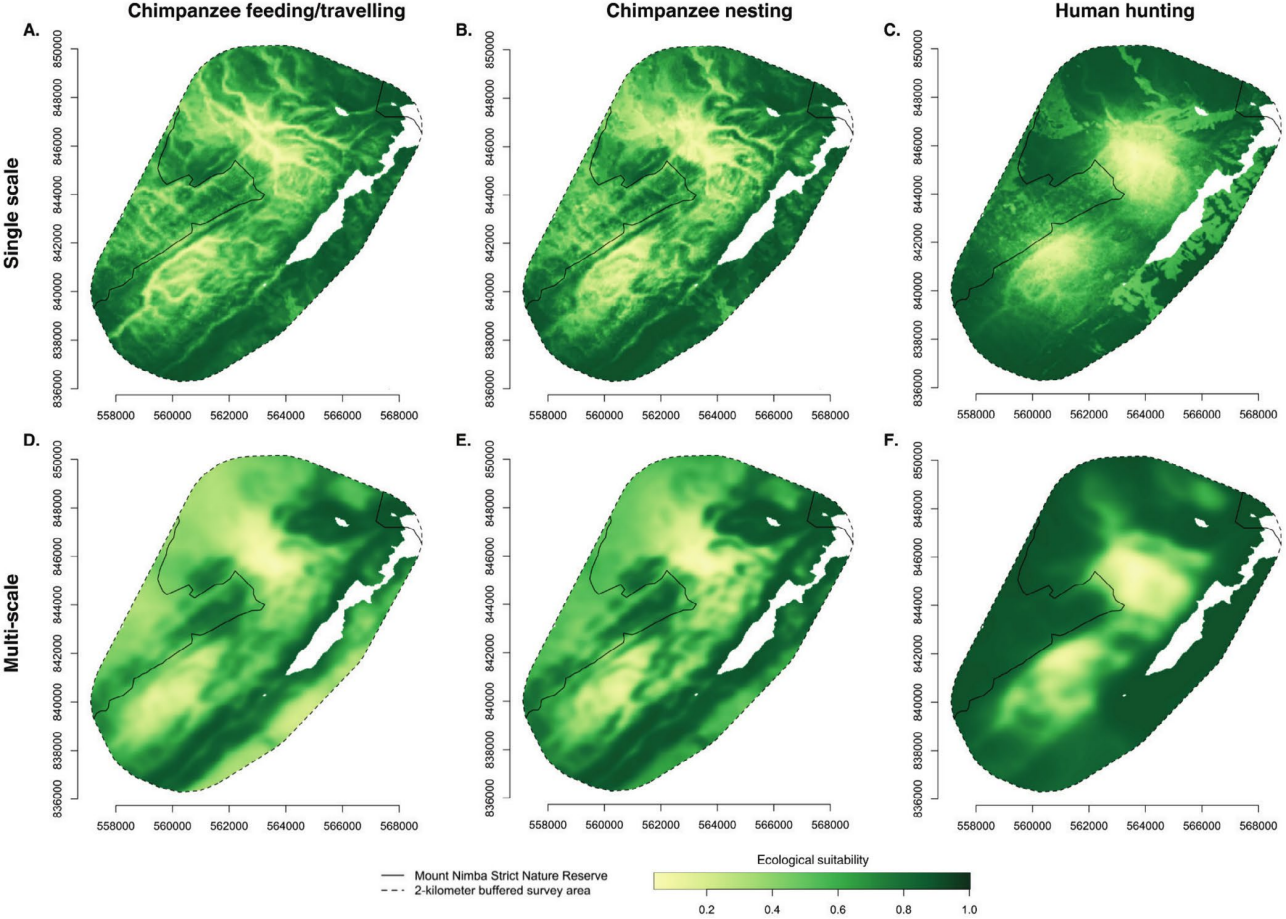
Predicted ecological suitability maps based on the 100 averaged GLM for each activity type, using single‐scale variables (top) and multi‐scale variables (bottom). Darker colours indicate higher values—dark green represents high ecological suitability, while lighter green to yellow represents lower ecological suitability. Predictions are masked outside the minimum convex polygon surrounding survey tracks expanded by a 2 km buffer, and above the highest surveyed elevation.

### 3.2 Variables and Scale Selection

Correlation analyses revealed high correlations among greenness, Enhanced Vegetation Index (EVI), and brightness. Only EVI was retained in all models. Similarly, roughness and slope were highly correlated, and only slope was retained in all models. A further correlation analysis conducted on optimally scaled variables for the GLM analyses identified high correlations between continuous heat‐insolation load index (CHILI) at 1500 m and slope at 1000 m in the hunting model, with the latter being retained (Figure S8). Additionally, overdispersion was assessed for all 600 averaged GLM analyses by computing the ratio of residual deviance to residual degrees of freedom; all values remained below 1.3, confirming the absence of overdispersion.

When selecting scales for the MS variables, the scales retained in the final chimpanzee feeding/travelling models were largely similar to those in the nesting models, with 7–8 variables selected at the same scale across the two chimpanzee activities. In contrast, between modelling approaches, the scales selected for BRT and GLM analyses showed less agreement, with only 2–3 variables selected at the same scales (Tables S2–S4).

Regarding overall variable selection and ranking, the dredge function (MuMIn R package) retained all predictors in GLM (Tables S7–S12). However, consistency in top‐ranked variables was limited between modelling approaches and across activity types (Tables S5 and S6). Distance to camp emerged as the most consistent predictor, ranking highly across nearly all activities in both modelling frameworks. Slope similarly demonstrated strong consistency. Elevation appeared as a key predictor for most chimpanzee activities, but only in BRT models. CTI ranked prominently in half the BRT models and in the SS GLM for feeding behaviour. The remaining variables exhibited substantial variation in importance across models and activities. In summary, distance to camp and slope showed consistent importance across models and activities, while elevation and CTI demonstrated model‐specific importance.

### 3.3 Human Hunting Patterns

For human hunting activities, distance to research camps was the only predictor with a 95% CI not overlapping zero in both hunting GLMs regardless of scale optimisation (SS: *β* = 1.69 × 10^−3^, 95% CI = [8.47 × 10^−4^, 2.53 × 10^−3^]; MS: *β* = 1.54 × 10^−3^, 95% CI = [4.95 × 10^−4^, 2.55 × 10^−3^]). In BRT models, distance to research camps was the most influential predictor in SS models (~35% relative influence; Figure 4C; Figures S5a and S5b) and second most influential in MS models (~19%; Figure 4F; Figure S6b). In MS models, tree cover at a 500 m scale had the highest relative influence (19.5%; Table S5), with hunting activity notably decreasing in areas where tree cover exceeded 90%.

**FIGURE 4:**
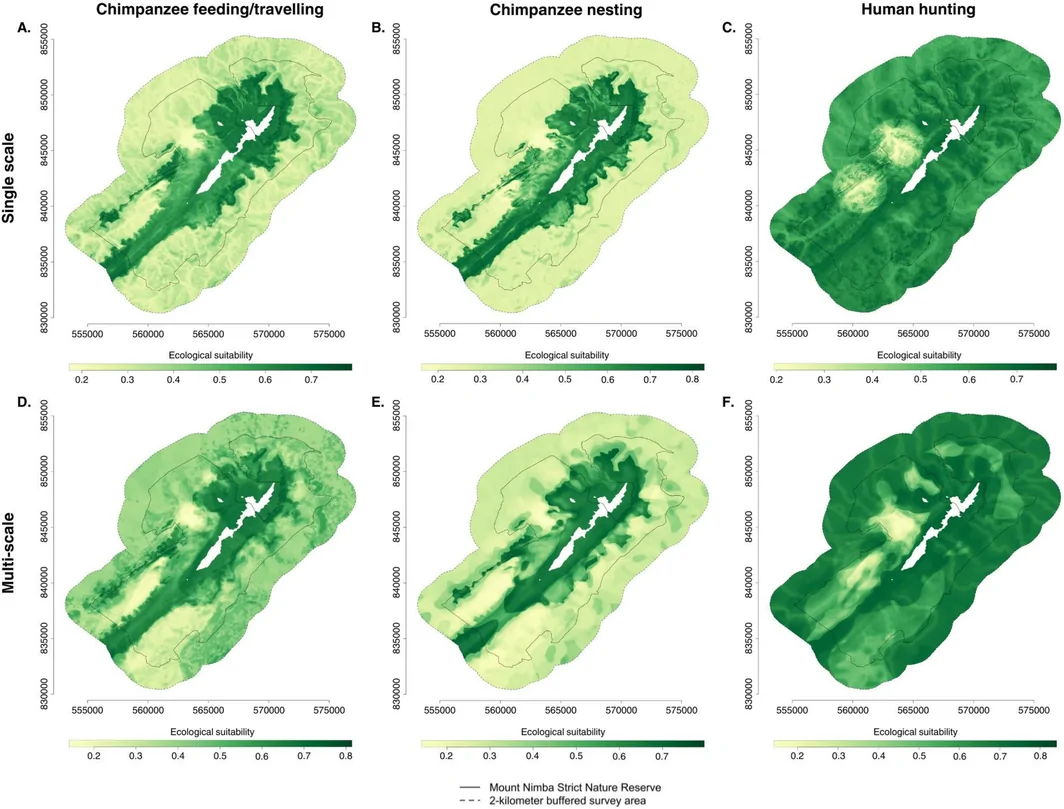
Predicted ecological suitability maps based on the 100 averaged BRT models for each activity type, using single‐scale variables (top) and multi‐scale variables (bottom). Darker colours indicate higher values—dark green represents high ecological suitability, while lighter green to yellow represents lower ecological suitability. Predictions are masked above the highest surveyed elevation and outside a 2 km buffer of the Mount Nimba Strict Nature Reserve boundary.

Hydrological factors were also important, with wetness at 1500 m ranking as the third most influential variable (14.5%), showing a negative relationship, and distance to rivers ranking prominently in both GLM and BRT modelling approaches (second in SS at ~10%; fourth in MS at 9.0%), with a positive relationship. Elevation ranked third in SS models, followed by compound topographic index (CTI; ~8%) and distance to roads (~6%; Figures S15, S7a and S7b). These findings suggested that accessibility and vegetation structure primarily shaped habitat use for hunting activities, with research camps acting as unintentional deterrents (Figures S5b and S6b).

### 3.4 Chimpanzee Feeding/Travelling Patterns

Chimpanzee feeding and travelling activities showed consistent associations with elevation, topographic variables, and vegetation characteristics. Elevation emerged as the most influential predictor across both BRT models (optimised at 30 m for MS models), demonstrating that chimpanzees prefer higher elevations for feeding and travelling. This preference was particularly pronounced at altitudes above 750 m (Figure 4; Figures S1b–S4b and S15).

Topographic Position Index (TPI) at 2000 m was the second most influential predictor (~12%) in MS feeding/travelling models, with increased ecological suitability observed at TPI values above −0.25, suggesting chimpanzees preferred using a range of topographical features, from lower‐lying areas to ridges at a landscape scale. The SS GLM also indicated a preference for ridges and higher slopes (CTI: *β* = −0.286, 95% CI = [−0.464, −0.108]; Slope: *β* = 0.072, 95% CI = [0.029, 0.114]), while the MS GLM highlighted a preference for warmer south‐facing slopes (Aspect: *β* = 0.032, 95% CI = [0.006, 0.051]) and steeper slopes (Slope: *β* = 0.105, 95% CI = [0.046, 0.160]). The MS approach further revealed that chimpanzees responded to warm slopes at a large scale (2 km) while selecting steeper slopes at a medium scale (250 m; Table 1). Slope was consistently important across all feeding/travelling models.

Vegetation quality, measured through EVI, showed a clear positive correlation with feeding/travelling suitability in the MS BRT models at 100 m scale (~9% relative influence), emphasising the role of dense, healthy vegetation in supporting these activities (Figures S2a, S7a and S15). Notably, distance to camp consistently ranked among the top four predictors across all chimpanzee feeding/travelling models, with suitability increasing beyond 1250 m from research camps in the BRT approach, suggesting some degree of human avoidance.

Finally, distance to roads ranked third in the SS BRT feeding models (~11%), with highest ecological suitability between zero and 2500 m and beyond 4500 m. Whereas distance to villages appeared only once among top five predictors (fourth in SS BRT feeding models; ~7.5%), with suitability decreasing as distance from villages increased (Figures S1a and S15).

### 3.5 Chimpanzee Nesting Patterns

Chimpanzee nesting displayed slightly different environmental preferences compared to feeding and travelling patterns, emphasising the distinct requirements for this activity. Slope was key in both GLM and BRT analyses, optimised at 250 m in MS GLM and showing high relative influence at 500 m scale (~18%) in MS BRT models. Response curves revealed a threshold effect at 15 degrees, above which nesting suitability increased substantially (Figures S3a and S15).

Elevation remained the most influential variable in BRT models (optimised at 100 m for MS approach), while TPI at 2000 m (~9.8%) and wetness at 2000 m (~11%) featured prominently (Table S6), indicating that landscape‐level topographic and hydrological features play an important role in nesting site selection. TPI at 2 km showed a tipping point around −0.3, with occurrences increasing beyond this threshold (Figures S4a, S7b and S15), suggesting that elevated areas relative to their surroundings were more favourable for chimpanzee nesting.

Regarding vegetation, EVI ranked fourth in SS BRT nesting models (~10%), while CTI appeared fifth in both SS and MS nesting models (~7% and ~9% respectively), with an optimised scale at 500 m. Distance to camp showed a clear positive association with nesting suitability, with 95% CI not overlapping zero, in nearly all models except MS BRT models (SS GLM: *β* = 1.11 × 10^−3^, 95% CI = [6.22 × 10^−4^, 1.60 × 10^−3^]; MS GLM: *β* = 9.38 × 10^−4^, 95% CI = [3.06 × 10^−4^, 1.54 × 10^−3^]), suggesting that chimpanzees tended to nest further from research camps (Figures 3 and 4; Tables S7–S12).

### 3.6 Spatial Overlap of Illegal Hunting and Chimpanzee Distribution

The prediction maps illustrating the differences in ecological suitability between chimpanzee and human models revealed that the western part of the study area exhibits lower chimpanzee ecological suitability and higher probability of human hunting activities (Figure 5; Figures S16–S19). Additionally, chimpanzee nesting behaviour showed more pronounced variations compared to feeding and travelling behaviours (Figures 3B and 4B). Indeed, areas highly unsuitable or highly suitable for feeding/travelling might be extremely unsuitable or extremely suitable for nesting, highlighting the sensitivity of nesting behaviour to ecological conditions. This sensitivity is evident in the difference maps, where the contrast in ecological suitability for nesting is more extreme, as indicated by darker colours compared to the maps for feeding and travelling (Figures S16–S19).

**FIGURE 5:**
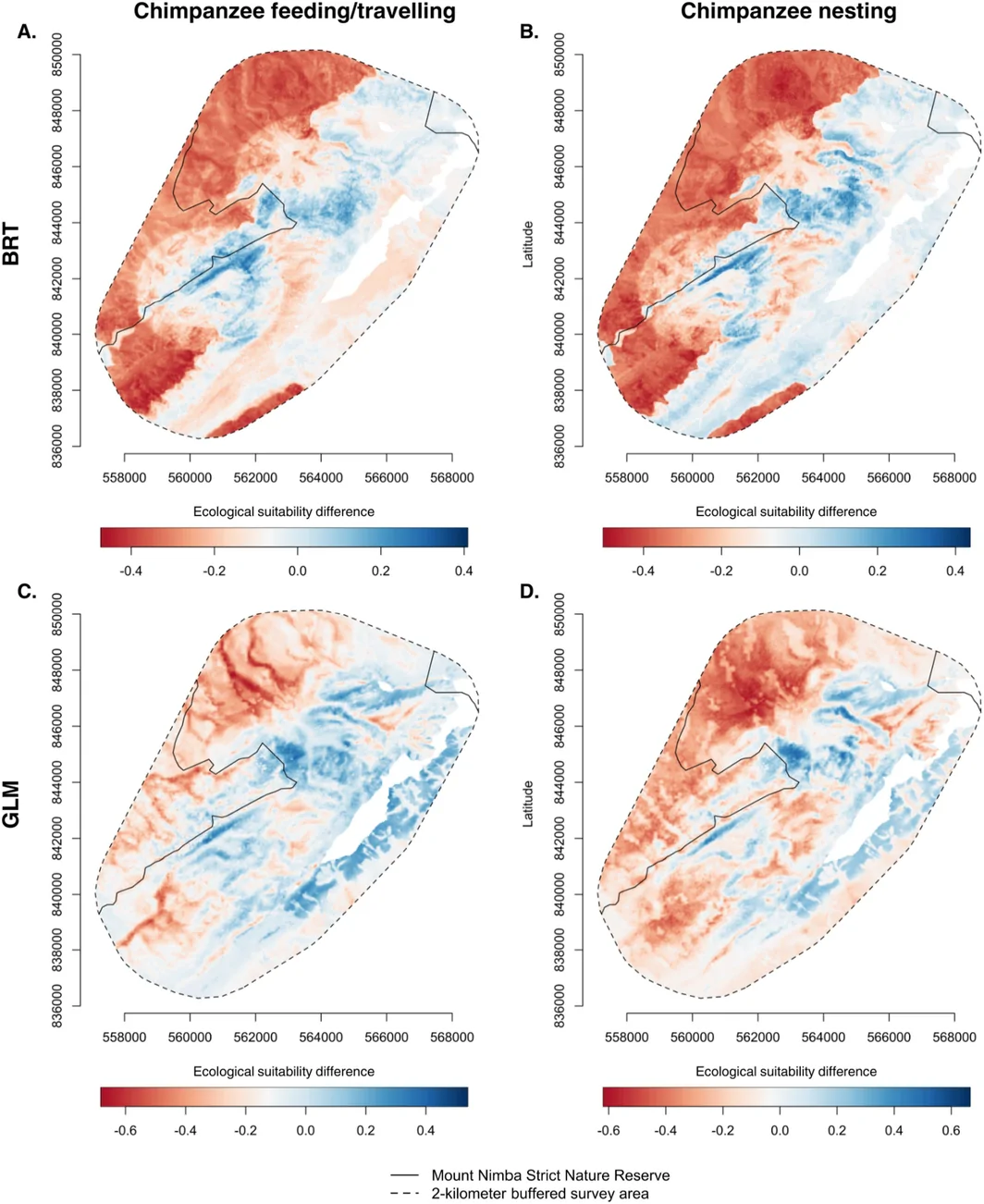
Maps illustrating the differences in ecological suitability estimated for the occurrence of chimpanzee feeding/travelling and human hunting (left), as well as for the chimpanzee nesting and human hunting (right). The results obtained with the BRT approach are depicted at the top, while those obtained with the GLM approach are reported at the bottom. Low values, represented by darker reds, indicate reduced ecological suitability for chimpanzee activities and/or increased suitability for human hunting, whereas high values, indicated by darker blues, reflect the opposite.

Correlation analyses between prediction surfaces revealed strong spatial associations between both chimpanzee activities and human hunting activities, particularly in SS GLM models (Table S13). The absolute average difference further supported that these predictive surfaces were relatively similar.

Although both chimpanzees and hunters generally avoided research camps, human hunting showed greater effect size (i.e., GLM regression coefficient *β*) in avoiding these areas, resulting in a protective buffer around research camps for chimpanzees. Consequently, areas near research camps showed overall higher ecological suitability for chimpanzees and/or lower human hunting activities. This pattern is particularly pronounced in SS BRT maps (Figure 5; Figures S16 and S17). Moreover, overlap maps showed research camps as areas of low interaction, while high‐elevation areas showed greatest overlap between hunters and chimpanzees (Figure 6; Figures S20–S23).

**FIGURE 6:**
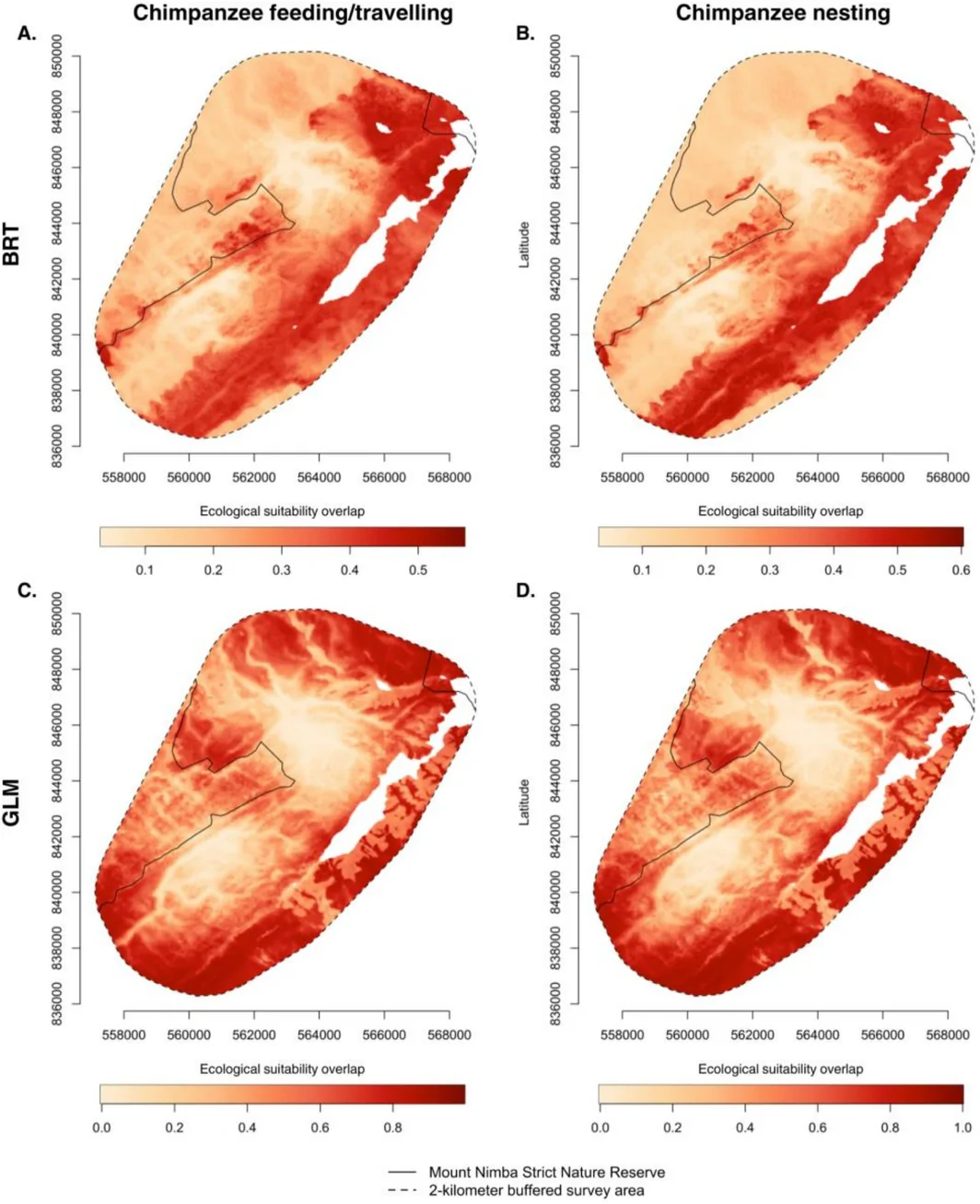
Maps illustrating the overlap in ecological suitability estimated for the occurrence of chimpanzee feeding/travelling and human hunting (left), as well as for the chimpanzee nesting and human hunting (right). The results obtained with the BRT approach are depicted at the top, while those obtained with the GLM approach are reported at the bottom. Higher values, represented by darker reds, indicate a greater likelihood of interaction between hunting activities and either chimpanzee feeding/travelling activities (left) or chimpanzee nesting sites (right).

In summary, correlation analyses indicated partial overlap in the use of the reserve by chimpanzees and hunters, while spatial analyses revealed distinct niche usage: western lowlands exhibited high hunting pressure but low chimpanzee suitability, high‐elevation zones showed significant overlap, and areas near research camps showed a relative reduction in predicted hunting activity that exceeded the reduction in predicted chimpanzee suitability, creating a differential buffer effect around camps.

## 4 Discussion

We examined behavioural patterns and spatial overlap between chimpanzee activities and illegal hunting in the Nimba Mountains in Guinea, finding that while chimpanzees and hunters in part select similar areas, chimpanzees demonstrate complex habitat preferences influenced by topography and vegetation density. Moreover, research camps provide potential protective buffer zones as they are more strongly avoided by hunters than by chimpanzees.

### 4.1 Modelling Approaches

Overall, the MS BRT models demonstrated slightly better predictive performance compared to their SS counterparts. Similarly, the MS GLM analyses for hunting exhibited better predictive performance than the SS version. However, the SS GLM for chimpanzees underperformed compared to the MS models. This is in line with previous studies across various species demonstrating the superiority of MS models (Timm et al. 2016; Boscolo and Metzger 2009; Kuhn et al. 2011, but see Martin and Fahrig 2012 for an exception). The lower cv AUC values observed for the BRT models compared to the GLM could be related to the use of a spatial cross‐validation when training the BRT models. Spatial cross‐validation, unlike standard cross‐validation, is designed to reduce overfitting caused by spatial autocorrelation, often resulting in more conservative but realistic performance estimates.

The two modelling approaches also yielded divergent spatial predictions, which warrants explicit discussion. First, GLM imposes a linear additive structure on the logit scale and uses model averaging over all candidate predictors weighted by AICc, whereas BRT captures non‐linear relationships and threshold effects between predictors, making it more flexible but less directly interpretable. Second, the different masking extents applied to each approach, reflecting their different extrapolation behaviours, contribute to visual differences between Figures 3 and 4. Third, as noted above, AUC estimates are not directly comparable across approaches given the different cross‐validation strategies employed. Rather than treating one approach as superior, both are retained as complementary: GLM analyses provide inferential clarity through model‐averaged coefficients and relative variable importance, while BRT analyses better capture non‐linear species‐environment relationships and offer more reliable predictions beyond the surveyed area.

Similar to several previous studies of various taxa (Thompson and McGarigal 2002; Macdonald et al. 2019; Ash et al. 2021), our study showed that estimated ecological suitability for chimpanzees is influenced by multiple factors across a range of spatial scales. The impact of spatial scale varied between different habitat components and within the same component (e.g., increased selection at larger scales for certain variables). Some environmental variables displayed distinctive scale‐dependent patterns, including critical thresholds and MS selection (e.g., TPI at 2000 m for chimpanzees and wetness at 1500 m for hunters). These findings highlight the importance of incorporating MS approaches into conservation planning, as scale‐dependent habitat features that may be overlooked by SS analyses can prove critical for identifying and protecting key areas for threatened species.

### 4.2 Chimpanzee Nesting Patterns

Slope emerged as a particularly influential factor in predicting chimpanzee nesting site distribution. Both scale approaches consistently indicated a preference for steeper slopes. A preference for steeper slopes may provide greater protection from predators and human disturbances, offering a more secure nesting environment. Fotang et al. (2021) indeed found that chimpanzees prefer nesting in tall trees on steep slopes, likely to avoid human hunters. Additionally, steeper areas may provide improved environmental conditions, including better drainage and reduced undergrowth density, facilitating nest construction. These findings align with prior research by Hernandez‐Aguilar (2009) and Dutton et al. (2016), which similarly highlighted chimpanzees' specific nesting site preferences.

Distance to research camps emerged as another important predictor, with chimpanzees consistently choosing nesting locations farther from research camps. EVI also played a crucial role, with higher values correlating with increased nesting ecological suitability, reflecting the importance of photosynthetically active vegetation (Campbell and Wynne 2011). Research by Koops, McGrew, de Vries, and Matsuzawa (2012) and Koops, McGrew, Matsuzawa, and Knapp (2012) supports this, showing that Seringbara chimpanzees favour large trees with dense foliage in primary forest for nesting. The CTI further revealed that intermediate values of topographic complexity were preferred for nesting, suggesting that balanced topographic features are more suitable.

Elevation proved to be another crucial predictor, with BRT models indicating high ecological suitability for nesting activities above approximately 750 m. This elevation threshold can be explained by the landscape's demographic and ecological characteristics. All nearby villages and most cultivated fields are situated below 700 m (Fitzgerald et al. 2018), while the protected status, remoteness, and extreme terrain of higher elevations likely hinder some types of human activities within the MNSNR. However, significant levels of hunting still occur at higher altitudes. Previous research by Koops, McGrew, de Vries, and Matsuzawa (2012) showed Seringbara chimpanzees' preference for nesting above 1000 m. Above approximately 1200–1300 m, the landscape transitions to high‐altitude grasslands, which generally offer limited resources for chimpanzees (Koops 2011). The relationship between elevation and predicted suitability for nesting suggests that chimpanzees are likely selecting nesting sites that are both secure and offer suitable microhabitats.

### 4.3 Chimpanzee Habitat Use for Feeding and Travelling

Chimpanzee feeding and travelling ecological suitability was influenced by several key environmental variables. Distance to camp emerged as a consistent predictor across all models, with chimpanzees tending to avoid areas closer to research camps, reflecting a broader tendency to steer clear of human presence. However, as both camps are situated at relatively low elevations (~700 m), this pattern may partly reflect a preference for higher‐altitude areas, which could appear as camp avoidance. Aspect also played a role, with chimpanzees preferring warmer south‐facing slopes.

EVI consistently showed a pattern across most BRT chimpanzee models, particularly in the feeding/travelling MS models, where it ranked as the third most influential variable at a 100 m scale. This indicates that chimpanzees prefer forested areas with dense, robust vegetation (Fitzgerald et al. 2018). Additionally, the key tree species used by chimpanzees for feeding predominantly thrive in primary forests (Koops 2011), which are associated with higher EVI values.

Topographic variables provided additional insights into chimpanzee habitat use. Slope showed a positive relationship with feeding and travelling ecological suitability, suggesting that steeper slopes were preferred for these activities. TPI at 2000 m scale showed a strong positive association with chimpanzee habitat use, with increased suitability in areas where TPI values exceeded −0.3. This finding suggests a preference for locations elevated relative to their surroundings, likely as a strategy to avoid valleys or depressions and potentially to gain better visibility. Research by Lemoine et al. (2023) found that chimpanzees are more likely to use hills near territorial borders to gather information about rivals, aligning with the observed large‐scale response to topographic features. Similarly, Gregory et al. (2014) observed that bearded saki monkeys favour ridges to reduce the energetic costs of travel and potentially aid in route‐based mental mapping. The use of topography for navigation and movement planning has not yet been investigated in depth for chimpanzees in the MNSNR (but see Green et al. 2020).

### 4.4 Illegal Hunting and Chimpanzee Habitat Overlap

Models of illegal hunting activities revealed several critical predictors, including distance to research camps, tree cover, elevation, and proximity to rivers. Tree cover emerged as the most influential variable in MS hunting models, particularly at 500 m scale. Areas with over 90% tree cover showed a reduction in predicted suitability for hunting activities, likely because dense forests present obstacles along with limited visibility to hunters. This pattern might reflect hunters' preference for more accessible, less densely vegetated areas, which are easier to navigate and more likely to harbour prey species other than chimpanzees. For chimpanzees, densely forested areas could serve as refuges from hunting pressures.

Elevation plays a crucial role in hunter‐chimpanzee interactions. While it is the third most influential variable in SS hunting models, it does not appear among the top five in MS models, indicating that hunters may not specifically target high elevations. Yet these areas still present significant overlap between chimpanzee and human activities, especially in the highest‐elevation zones. These zones, while providing security from other human disturbances, are also where encounters between chimpanzees and hunters are most likely. This finding underscores the need for intensive monitoring in high‐altitude regions to mitigate potential conflicts.

Wetness at 1500 m scale and proximity to rivers also influenced human hunting behaviour. Wet environments and areas near rivers may deter hunters due to difficult terrain and potentially challenging crossing conditions. Additionally, we found that research camps might also create protective buffer zones for chimpanzees, as hunters tend to avoid these areas more strongly than chimpanzees do, resulting in higher chimpanzee suitability near research camps. Hence, these findings imply that research activities may inadvertently aid conservation by deterring hunters (Campbell et al. 2011).

The prediction maps revealed a contrast between areas of high chimpanzee ecological suitability and zones of intense hunting activity. The western portion of the study area consistently exhibited lower use by chimpanzees coupled with higher hunting probability, suggesting potential displacement effects. Chimpanzee nesting patterns demonstrated more pronounced sensitivity to environmental conditions compared to feeding or travelling, with suitability maps displaying more extreme spatial contrasts between these activities. This sensitivity likely reflects the greater importance of security considerations during vulnerable resting periods.

Model correlation coefficients and averaged absolute differences reveal that chimpanzees and hunters select and avoid similar areas overall, despite responding differently to individual predictors. The significant niche overlap suggests that chimpanzees may either tolerate human presence or employ temporal displacement strategies, avoiding areas during periods of hunting activity.

More critically, the observed spatial overlap may pose a long‐term threat to chimpanzees. Continued human activity in these areas could lead to habitat suppression, gradually forcing chimpanzees to retreat to increasingly less suitable environments. Conservation strategies must therefore prioritise high‐elevation areas and dense forests to mitigate human activities and protect critical chimpanzee habitats. The strategic positioning of research or monitoring infrastructure may provide localised protective effects for chimpanzee populations, while high‐elevation areas, emerging as zones of greatest human‐chimpanzee interaction potential, should be prioritised for intensive monitoring and targeted conservation interventions.

### 4.5 Future Directions

While our study can help to design conservation strategies for chimpanzees, it is crucial to acknowledge limitations. The research teams focused on chimpanzee behaviour, with GPS tracking targeting areas frequented by these apes, potentially biasing pseudo‐absences sampling. In the BRT models, data was projected onto areas where no direct observations were recorded, a common limitation in predictive ENM studies posing challenges in interpreting ecological suitability responses to predictor variables. Although there are no universal solutions to these challenges (Eger et al. 2017; Elith et al. 2010; Zurell et al. 2012), future research could mitigate them by extending surveys across the MNSNR. While we attempted to incorporate comprehensive fine‐scale variables, some pertinent variables (e.g., fruit availability) could not be included. The availability of high‐quality, fine‐scale layers remains a significant challenge. Further research could involve collecting additional data on anthropogenic disturbances, examining activities like slash‐and‐burn agriculture, and conducting ground‐truthing to validate predictions, potentially revealing nuances not captured by remote sensing or automated modelling alone.

To build on this foundation, it is essential to apply these insights to real‐world conservation strategies. Having identified areas where chimpanzee habitat preferences overlap with zones of high human activity, targeted management interventions can now be developed, including directed monitoring and patrolling efforts and the continued engagement of local communities in conservation initiatives. The integration of advanced technologies and spatial modelling in conservation planning offers a powerful toolset to prioritise efforts where they are needed most, ultimately contributing to the long‐term survival of chimpanzees in the MNSNR.

## 5 Conclusions

While our study is specific to the Seringbara study area in the Nimba Mountains, some of our findings may offer insights relevant to primate conservation in comparable montane contexts. First, topography's strong influence on habitat selection underscores the importance of preserving diverse landscape features rather than focusing solely on vegetation (Green et al. 2020). Second, the differential avoidance patterns of hunters and chimpanzees regarding research camps suggest that strategic placement of research and conservation infrastructure can create protective buffers for endangered primates; however, care must be taken not to establish research camps in areas most used by chimpanzees, as their general avoidance of humans might discourage them from visiting those areas despite the availability of resources. Third, our methodological approach demonstrates how MS ecological niche modelling can identify critical habitat features that might be overlooked by SS analyses, potentially improving habitat assessment protocols for threatened species worldwide. These findings contribute to our understanding of human‐wildlife spatial dynamics in the MNSNR and, if replicated across additional sites and taxonomic groups, may inform broader frameworks for evidence‐based conservation planning. Combined with proactive management and community engagement, spatial modelling approaches such as those applied here could support efforts toward the long‐term coexistence of humans and wildlife in critical habitats.

## Author Contributions

**Lucas Degrève:** conceptualization (equal), formal analysis (equal), investigation (equal), methodology (equal), project administration (equal), visualization (lead), writing – original draft (lead), writing – review and editing (equal). **Diana Erazo:** conceptualization (equal), formal analysis (equal), funding acquisition (equal), investigation (equal), methodology (equal), resources (equal), supervision (equal), validation (equal), writing – review and editing (equal). **Maegan Fitzgerald:** conceptualization (equal), data curation (equal), formal analysis (equal), funding acquisition (equal), investigation (equal), methodology (equal), resources (equal), supervision (equal), validation (equal), visualization (equal), writing – review and editing (equal). **Henry Didier Camara:** conceptualization (equal), data curation (equal), formal analysis (equal), investigation (equal), methodology (equal), resources (equal). **Gnan Mamy:** conceptualization (equal), data curation (equal), formal analysis (equal), investigation (equal), methodology (equal), resources (equal). **Simon Dellicour:** conceptualization (equal), formal analysis (equal), funding acquisition (equal), investigation (equal), methodology (equal), project administration (supporting), resources (equal), supervision (equal), validation (equal), visualization (supporting), writing – review and editing (equal). **Żaneta Kaszta:** conceptualization (equal), data curation (equal), formal analysis (equal), investigation (equal), methodology (equal), software (supporting), supervision (equal), validation (equal), visualization (supporting), writing – review and editing (equal). **Kathelijne Koops:** conceptualization (equal), data curation (equal), formal analysis (equal), funding acquisition (equal), investigation (equal), methodology (equal), project administration (supporting), resources (equal), supervision (equal), validation (equal), writing – review and editing (equal).

## Funding

Field research was supported by grants from the A.H. Schultz Foundation, the Jane Goodall Institute Schweiz, and the Jane Goodall Institute Germany to K.K. and M.F. Furthermore, K.K. was funded by a SNSF Eccellenza Professorial Fellowship (no. PCEFP3_186967). S.D. acknowledges support from the *Fonds National de la Recherche Scientifique* (F.R.S.‐FNRS, Belgium; grant no. F.4515.22), from the Research Foundation—Flanders (*Fonds voor Wetenschappelijk Onderzoek*—Vlaanderen, FWO, Belgium; grant no. G098321N), and from the European Union Horizon 2020 projects MOOD (grant agreement no. 874850) and LEAPS (grant agreement no. 101094685). D.E. acknowledges support from the European Union's Horizon 2020 research and innovation programme under the Marie Skłodowska‐Curie (grant agreement no. 801505), from the *Fonds National de la Recherche Scientifique* (F.R.S.‐FNRS, Belgium), and the Belgian Science Policy Office in the framework of the FED‐tWIN project RECORDED (Prf‐2021‐033).

## Conflicts of Interest

The authors declare no conflicts of interest. The authors declared all research support for this reported work in the ‘Acknowledgements’ section.

## Supporting information


**Table S1:** List of environmental variables selected for modelling purposes.
**Table S2:** Optimal scales for variables in the chimpanzee feeding models, determined by mean cross‐validated area under the receiver operating characteristic curve (cv AUC) for 100 independent analyses and 100 sets of pseudo‐absence points using univariate GLM and BRT modelling.
**Table S3:** Optimal scales for variables in the chimpanzee nesting model, determined by mean cross‐validated area under the receiver operating characteristic curve (cv AUC) from 100 runs and 100 pseudo‐absence sets using univariate GLM and BRT modelling.
**Table S4:** Optimal scales for variables in the human hunting model, determined by mean cross‐validated area under the receiver operating characteristic curve (cv AUC) from 100 runs and 100 pseudo‐absence sets using univariate GLM and BRT modelling.
**Table S5:** Predictor variables ranked by effect size *β* (standardised regression coefficients) per averaged binomial model trained with the GLM approach.
**Table S6:** Top five predictor variables ranked by relative importance (in percentage) per kind of model trained with the BRT approach.
**Table S7:** Summary of 100 averaged binomial GLM runs using the dredge function from the MuMIn package for the chimpanzee feeding model with single‐scale variables.
**Table S8:** Summary of 100 averaged binomial GLM runs using the dredge function from the MuMIn package for the chimpanzee feeding model with multi‐scale variables.
**Table S9:** Summary of 100 averaged binomial GLM runs using the dredge function from the MuMIn package for the chimpanzee nesting model with single‐scale variables.
**Table S10:** Summary of 100 averaged binomial GLM runs using the dredge function from the MuMIn package for the chimpanzee nesting model with multi‐scale variables.
**Table S11:** Summary of 100 averaged binomial GLM runs using the dredge function from the MuMIn package for the human hunting model with single‐scale variables.
**Table S12:** Summary of 100 averaged binomial GLM runs using the dredge function from the MuMIn package for the human hunting model with multi‐scale variables.
**Table S13:** Spatial correlation coefficients and averaged absolute differences between predicted chimpanzee activity (Model 1) and human hunting activity (Model 2) surfaces across modelling approaches (BRT, GLM) and scale methods (single‐scale vs. multi‐scale).
**Figure S1a:** Response curves of the 100 BRT models for the feeding model using single‐scale variables.
**Figure S1b:** Predicted ecological suitability map (left) and standard deviation map (right), based on the average of the 100 BRT models for the feeding model using single‐scale variables.
**Figure S2a:** Response curves of the 100 BRT models for the feeding model using scale‐optimised variables.
**Figure S2b:** Predicted ecological suitability map (left) and standard deviation map (right), based on the average of the 100 BRT models for the feeding model using scale‐optimised variables.
**Figure S3a:** Response curves of the 100 BRT models for the nesting model using single‐scale variables.
**Figure S3b:** Predicted ecological suitability map (left) and standard deviation map (right), based on the average of the 100 BRT models for the nesting model using single‐scale variables.
**Figure S4a:** Response curves of the 100 BRT models for the nesting model using scale‐optimised variables.
**Figure S4b:** Predicted ecological suitability map (left) and standard deviation map (right), based on the average of the 100 BRT models for the nesting model using scale‐optimised variables.
**Figure S5a:** Response curves of the 100 BRT models for the hunting model using single‐scale variables.
**Figure S5b:** Predicted ecological suitability map (left) and standard deviation map (right), based on the average of the 100 BRT models for the hunting model using single‐scale variables.
**Figure S6a:** Response curves of the 100 BRT models for the hunting model using scale‐optimised variables.
**Figure S6b:** Predicted ecological suitability map (left) and standard deviation map (right), based on the average of the 100 BRT models for the hunting model using scale‐optimised variables.
**Figure S7a:** Box plot of the predictor variables, ranked by percent contribution, for the chimpanzee feeding model using single‐scale variables (A), using scale‐optimised variables (B), and for the human hunting model using single‐scale variables (C).
**Figure S7b:** Box plot of the predictor variables, ranked by percent contribution, for the human hunting model using scale‐optimised variables (D), for the chimpanzee nesting model using single‐scale variables (E) and using scale‐optimised variables (F).
**Figure S8:** Plots of the correlation matrix of the different environmental variables that showed high correlation. Correlation coefficients were calculated using Spearman's correlation.
**Figure S9:** Predicted ecological suitability map (left) and standard deviation map (right), based on the 100 averaged GLM for the chimpanzee feeding model using single‐scale variables.
**Figure S10:** Predicted ecological suitability map (left) and standard deviation map (right), based on the 100 averaged GLM for the chimpanzee feeding model using scale‐optimised variables.
**Figure S11:** Predicted ecological suitability map (left) and standard deviation map (right), based on the 100 averaged GLM for the human hunting model using single‐scale variables.
**Figure S12:** Predicted ecological suitability map (left) and standard deviation map (right), based on the 100 averaged GLM for the human hunting model using scale‐optimised variables.
**Figure S13:** Predicted ecological suitability map (left) and standard deviation map (right), based on the 100 averaged GLM for the human hunting model using single‐scale variables.
**Figure S14:** Predicted ecological suitability map (left) and standard deviation map (right), based on the 100 averaged GLM for the human hunting model using scale‐optimised variables.
**Figure S15:** Response curves for the five most influential predictor variables, ranked by their relative importance (in percentage) in 100 replicate BRT models trained for each activity type.
**Figure S16:** Map illustrating the differences in ecological suitability between the BRT chimpanzee feeding/travelling model and the BRT human hunting model using single‐scale variables (left) and multi‐scale variables (right).
**Figure S17:** Map illustrating the differences in ecological suitability between the BRT chimpanzee nesting model and the BRT human hunting model using single‐scale variables (left) and multi‐scale variables (right).
**Figure S18:** Map illustrating the differences in ecological suitability between the GLM chimpanzee feeding/travelling model and the GLM human hunting model using single‐scale variables (left) and multi‐scale variables (right).
**Figure S19:** Map illustrating the differences in ecological suitability between the GLM chimpanzee nesting model and the GLM human hunting model using single‐scale variables (left) and multi‐scale variables (right).
**Figure S20:** Map showing areas of overlap between the BRT chimpanzee feeding/travelling model and the BRT human hunting model using single‐scale variables (left) and multi‐scale variables (right).
**Figure S21:** Map showing areas of overlap between the BRT chimpanzee nesting model and the BRT human hunting model using single‐scale variables (left) and multi‐scale variables (right).
**Figure S22:** Map showing areas of overlap between the GLM chimpanzee feeding/travelling model and the GLM human hunting model using single‐scale variables (left) and multi‐scale variables (right).
**Figure S23:** Map showing areas of overlap between the GLM chimpanzee nesting model and the GLM human hunting model using single‐scale variables (left) and multi‐scale variables (right).

## Data Availability

Environmental layers, and R scripts used for modelling are openly available. The full codebase is provided via GitHub, ensuring full reproducibility of our analyses: github.com/ldegrev/Seringbara. Species occurrence data are uploaded for review purposes only, as the western chimpanzee ( *Pan troglodytes verus* ) is listed as Critically Endangered on the IUCN Red List of Threatened Species. All the required data are uploaded as Supporting Information.

## References

[ece374384-bib-0002] Angel, F. 1943. La vie des Caméléons et Autres Lézards. FeniXX.

[ece374384-bib-0003] Ash, E. , D. W. Macdonald , S. A. Cushman , A. Noochdumrong , T. Redford , and Ż. Kaszta . 2021. “Optimization of Spatial Scale, but Not Functional Shape, Affects the Performance of Habitat Suitability Models: A Case Study of Tigers ( *Panthera tigris* ) in Thailand.” Landscape Ecology 36: 455–474. 10.1007/s10980-020-01105-6.

[ece374384-bib-0004] Austin, M. P. 1987. “Models for the Analysis of Species' Response to Environmental Gradients.” In Theory and Models in Vegetation Science: Proceedings of Symposium, Uppsala, July 8–13, 1985, 35–45. Springer Netherlands. 10.1007/978-94-009-4061-1_4.

[ece374384-bib-0006] Barbet‐Massin, M. , F. Jiguet , C. H. Albert , and W. Thuiller . 2012. “Selecting Pseudo‐Absences for Species Distribution Models: How, Where and How Many?” Methods in Ecology and Evolution 3, no. 2: 327–338. 10.1111/j.2041-210X.2011.00172.x.

[ece374384-bib-0007] Barton, K. 2024. “Package—MuMIn: Multi Model Inference.” R Package Version 1.48.4.

[ece374384-bib-0008] Bjørnstad, O. N. 2023. “Package Ncf: Spatial Covariance Functions.” R Package Version 1.3‐3.

[ece374384-bib-0009] Boscolo, D. , and J. P. Metzger . 2009. “Is Bird Incidence in Atlantic Forest Fragments Influenced by Landscape Patterns at Multiple Scales?” Landscape Ecology 24: 907–918. 10.1007/s10980-009-9370-8.

[ece374384-bib-0010] Bryson‐Morrison, N. , J. Tzanopoulos , T. Matsuzawa , and T. Humle . 2017. “Activity and Habitat Use of Chimpanzees ( *Pan troglodytes verus* ) in the Anthropogenic Landscape of Bossou, Guinea, West Africa.” International Journal of Primatology 38: 282–302. 10.1007/s10764-016-9947-4. PMC5422491 28546651

[ece374384-bib-0011] Burnham, K. P. , and D. R. Anderson , eds. 2002. Model Selection and Multimodel Inference: A Practical Information‐Theoretic Approach. Springer New York. 10.1007/b97636.

[ece374384-bib-0012] Campbell, G. , and M. Houngbedji . 2015. “Conservation Status of the West African Chimpanzee (*Pan troglodytes verus*) in Togo and Benin.” Report to Primate Action Fund, Arlington, VA. http://www.primate‐sg.org/unpublished‐reports.

[ece374384-bib-0013] Campbell, G. , H. Kuehl , A. Diarrassouba , P. K. N'Goran , and C. Boesch . 2011. “Long‐Term Research Sites as Refugia for Threatened and Over‐Harvested Species.” Biology Letters 7, no. 5: 723–726. 10.1098/rsbl.2011.0155. PMC3169048 21450724

[ece374384-bib-0014] Campbell, J. B. , and R. H. Wynne . 2011. Introduction to Remote Sensing. Guilford Press. 10.3390/rs5010282.

[ece374384-bib-0015] Chiaverini, L. , D. W. Macdonald , A. J. Hearn , et al. 2023. “Not Seeing the Forest for the Trees: Generalised Linear Model Out‐Performs Random Forest in Species Distribution Modelling for Southeast Asian Felids.” Ecological Informatics 75: 102026. 10.1016/j.ecoinf.2023.102026.

[ece374384-bib-0017] Dutton, P. , E. Moltchanova , and H. Chapman . 2016. “Nesting Ecology of a Small Montane Population of the Nigerian/Cameroon Chimpanzee ( *Pan troglodytes ellioti* ) in Nigeria.” Folia Primatologica 87, no. 6: 361–374. 10.1159/000454921. 28110325

[ece374384-bib-0018] Eger, A. M. , J. M. Curtis , M. J. Fortin , I. M. Côté , and F. Guichard . 2017. “Transferability and Scalability of Species Distribution Models: A Test With Sedentary Marine Invertebrates.” Canadian Journal of Fisheries and Aquatic Sciences 74, no. 5: 766–778. 10.1139/cjfas-2016-0129.

[ece374384-bib-0019] Elith, J. , C. H. Graham , R. P. Anderson , et al. 2006. “Novel Methods Improve Prediction of Species' Distributions From Occurrence Data.” Ecography 29: 129–151. 10.1111/j.2006.0906-7590.04596.x.

[ece374384-bib-0020] Elith, J. , M. Kearney , and S. Phillips . 2010. “The Art of Modelling Range‐Shifting Species.” Methods in Ecology and Evolution 1, no. 4: 330–342. 10.1111/j.2041-210X.2010.00036.x.

[ece374384-bib-0021] Elith, J. , and J. R. Leathwick . 2009. “Species Distribution Models: Ecological Explanation and Prediction Across Space and Time.” Annual Review of Ecology, Evolution, and Systematics 40: 677–697. 10.1146/annurev.ecolsys.110308.120159.

[ece374384-bib-0022] Elith, J. , J. R. Leathwick , and T. Hastie . 2008. “A Working Guide to Boosted Regression Trees.” Journal of Animal Ecology 77: 802–813. 10.1111/j.1365-2656.2008.01390.x. 18397250

[ece374384-bib-0023] Fitzgerald, M. , R. Coulson , A. M. Lawing , T. Matsuzawa , and K. Koops . 2018. “Modeling Habitat Suitability for Chimpanzees ( *Pan troglodytes verus* ) in the Greater Nimba Landscape, Guinea, West Africa.” Primates 59: 361–375. 10.1007/s10329-018-0657-8. 29524002

[ece374384-bib-0024] Fotang, C. , U. Bröring , C. Roos , et al. 2021. “Environmental and Anthropogenic Effects on the Nesting Patterns of Nigeria–Cameroon Chimpanzees in North‐West Cameroon.” American Journal of Primatology 83, no. 9: e23312. 10.1002/ajp.23312. 34343361

[ece374384-bib-0025] Furuichi, T. , C. Hashimoto , and Y. Tashiro . 2001. “Extended Application of a Marked‐Nest Census Method to Examine Seasonal Changes in Habitat Use by Chimpanzees.” International Journal of Primatology 22: 913–928. 10.1023/A:1012057403512.

[ece374384-bib-0026] Ginn, L. P. , J. Robison , I. Redmond , and K. A. I. Nekaris . 2013. “Strong Evidence That the West African Chimpanzee Is Extirpated From Burkina Faso.” Oryx 47: 325–326. 10.1017/S0030605313000434.

[ece374384-bib-0027] Gorelick, N. , M. Hancher , M. Dixon , S. Ilyushchenko , D. Thau , and R. Moore . 2017. “Google Earth Engine: Planetary‐Scale Geospatial Analysis for Everyone.” Remote Sensing of Environment 202: 18–27.

[ece374384-bib-0028] Green, S. J. , B. J. Boruff , and C. C. Grueter . 2020. “From Ridge Tops to Ravines: Landscape Drivers of Chimpanzee Ranging Patterns.” Animal Behaviour 163: 51–60. 10.1016/j.anbehav.2020.02.016.

[ece374384-bib-0029] Gregory, T. , A. Mullett , and M. A. Norconk . 2014. “Strategies for Navigating Large Areas: A GIS Spatial Ecology Analysis of the Bearded Saki Monkey, *Chiropotes sagulatus* , in Suriname.” American Journal of Primatology 76, no. 6: 586–595. 10.1002/ajp.22251. 24375420

[ece374384-bib-0030] Guillaumet, J. L. , and E. Adjanohoun . 1971. “Le Milieu Naturel de la Côte‐d'Ivoire.” Mémoires ORSTOM 50: 157–264.

[ece374384-bib-0031] Guisan, A. , T. C. Edwards Jr. , and T. Hastie . 2002. “Generalized Linear and Generalized Additive Models in Studies of Species Distributions: Setting the Scene.” Ecological Modelling 157, no. 2–3: 89–100. 10.1016/S0304-3800(02)00204-1.

[ece374384-bib-0032] Guisan, A. , and W. Thuiller . 2005. “Predicting Species Distribution: Offering More Than Simple Habitat Models.” Ecology Letters 8, no. 9: 993–1009. 10.1111/j.1461-0248.2005.00792.x. 34517687

[ece374384-bib-0033] Guisan, A. , W. Thuiller , and N. E. Zimmermann . 2017. Habitat Suitability and Distribution Models: With Applications in R. Cambridge University Press.

[ece374384-bib-0035] Heinicke, S. , R. Mundry , C. Boesch , et al. 2019. “Advancing Conservation Planning for Western Chimpanzees Using IUCN SSC A.P.E.S.–the Case of a Taxon‐Specific Database.” Environmental Research Letters 14: 064001. 10.1088/1748-9326/ab1379.

[ece374384-bib-0036] Heinicke, S. , R. Mundry , C. Boesch , et al. 2019. “Towards Systematic and Evidence‐Based Conservation Planning for Western Chimpanzees.” American Journal of Primatology 81, no. 9: e23042. 10.1002/ajp.23042. 31468565

[ece374384-bib-0037] Hernandez‐Aguilar, R. A. 2009. “Chimpanzee Nest Distribution and Site Reuse in a Dry Habitat: Implications for Early Hominin Ranging.” Journal of Human Evolution 57, no. 4: 350–364. 10.1016/j.jhevol.2009.03.007. 19744699

[ece374384-bib-0038] Hijmans, R. J. , S. Phillips , J. Leathwick , J. Elith , and M. R. J. Hijmans . 2023. “Package ‘dismo’.” R Package Version 1.3‐14.

[ece374384-bib-0040] Humle, T. 2003. “*Culture and Variation in Wild Chimpanzee Behaviour: A Study of Three Communities in West Africa*.” Ph.D. thesis, University of Stirling.

[ece374384-bib-0041] Humle, T. , C. Boesch , G. Campbell , et al. 2016. “*Pan troglodytes ssp. verus*.” The IUCN Red List of Threatened Species 2016: e.T15935A17989872. 10.2305/IUCN.UK.2016-2.RLTS.T15935A17989872.en.

[ece374384-bib-0042] IUCN SSC Primate Specialist Group . 2020. “Regional Action Plan for the Conservation of Western Chimpanzees (*Pan troglodytes verus*) 2020–2030.” *IUCN, International Union for Conservation of Nature*, 1–72. 10.2305/IUCN.CH.2020.SSC-RAP.2.en.

[ece374384-bib-0043] Jiménez‐Valverde, A. 2012. “Insights Into the Area Under the Receiver Operating Characteristic Curve (AUC) as a Discrimination Measure in Species Distribution Modelling.” Global Ecology and Biogeography 21, no. 4: 498–507. 10.1111/j.1466-8238.2011.00683.x.

[ece374384-bib-0044] Jiménez‐Valverde, A. 2014. “Threshold‐Dependence as a Desirable Attribute for Discrimination Assessment: Implications for the Evaluation of Species Distribution Models.” Biodiversity and Conservation 23: 369–385. 10.1007/s10531-013-0606-1.

[ece374384-bib-0045] Koops, K. 2011. “*Elementary Technology of Foraging and Shelter in the Chimpanzees* (*Pan troglodytes verus*) *of the Nimba Mountains*, *Guinea*.” PhD thesis, University of Cambridge.

[ece374384-bib-0046] Koops, K. , T. Humle , P. Frandsen , et al. 2023. “Genetics as a Novel Tool in Mining Impact Assessment and Biomonitoring of Critically Endangered Western Chimpanzees in the Nimba Mountains, Guinea.” Conservation Science and Practice 5, no. 4: e12898. 10.1111/csp2.12898.

[ece374384-bib-0047] Koops, K. , W. C. McGrew , H. de Vries , and T. Matsuzawa . 2012. “Nest‐Building by Chimpanzees ( *Pan troglodytes verus* ) at Seringbara, Nimba Mountains: Antipredation, Thermoregulation, and Antivector Hypotheses.” International Journal of Primatology 33: 356–380. 10.1007/s10764-012-9585-4.

[ece374384-bib-0049] Koops, K. , W. C. McGrew , T. Matsuzawa , and L. A. Knapp . 2012. “Terrestrial Nest‐Building by Wild Chimpanzees ( *Pan troglodytes* ): Implications for the Tree‐to‐Ground Sleep Transition in Early Hominins.” American Journal of Physical Anthropology 148, no. 3: 351–361. 10.1016/j.anbehav.2012.10.022. 22460549

[ece374384-bib-0050] Kormos, R. , C. Boesch , M. I. Bakarr , and T. M. Butynski . 2003. West African Chimpanzees: Status Survey and Conservation Action Plan. International Union for Conservation of Nature and Natural Resources. https://portals.iucn.org/library/sites/library/files/documents/2003‐059.pdf.

[ece374384-bib-0051] Kortlandt, A. 1986. “The Use of Stone Tools by Wild‐Living Chimpanzees and Earliest Hominids.” Journal of Human Evolution 15, no. 2: 77–132. 10.4159/harvard.9780674183858.c17.

[ece374384-bib-0052] Kühl, H. S. , C. Boesch , L. Kulik , et al. 2019. “Human Impact Erodes Chimpanzee Behavioral Diversity.” Science 363, no. 6434: 1453–1455. 10.1126/science.aau4532. 30846610

[ece374384-bib-0053] Kühl, H. S. , T. Sop , E. A. Williamson , et al. 2017. “The Critically Endangered Western Chimpanzee Declines by 80%.” American Journal of Primatology 79, no. 9. 10.1002/ajp.22681. 28671715

[ece374384-bib-0054] Kuhn, A. , J. Copeland , J. Cooley , et al. 2011. “Modeling Habitat Associations for the Common Loon ( *Gavia immer* ) at Multiple Scales in Northeastern North America.” Avian Conservation and Ecology 6, no. 1: 4. 10.5751/ACE-00451-060104.

[ece374384-bib-0055] Lemoine, S. R. , L. Samuni , C. Crockford , and R. M. Wittig . 2023. “Chimpanzees Make Tactical Use of High Elevation in Territorial Contexts.” PLoS Biology 21, no. 11: e3002350. 10.1371/journal.pbio.3002350. PMC10621857 37917608

[ece374384-bib-0056] Levin, S. A. 1992. “The Problem of Pattern and Scale in Ecology: The Robert H. MacArthur Award Lecture.” Ecology 73, no. 6: 1943–1967. 10.2307/1941447.

[ece374384-bib-0057] Lobo, J. M. , A. Jiménez‐Valverde , and R. Real . 2008. “AUC: A Misleading Measure of the Performance of Predictive Distribution Models.” Global Ecology and Biogeography 17, no. 2: 145–151. 10.1111/j.1466-8238.2007.00358.x.

[ece374384-bib-0058] Macdonald, D. W. , H. M. Bothwell , Ż. Kaszta , et al. 2019. “Multi‐Scale Habitat Modelling Identifies Spatial Conservation Priorities for Mainland Clouded Leopards ( *Neofelis nebulosa* ).” Diversity and Distributions 25, no. 10: 1639–1654. 10.1111/ddi.12967.

[ece374384-bib-0059] Martin, A. E. , and L. Fahrig . 2012. “Measuring and Selecting Scales of Effect for Landscape Predictors in Species–Habitat Models.” Ecological Applications 22, no. 8: 2277–2292. 10.1890/11-2224.1. 23387125

[ece374384-bib-0060] Mateo Sanchez, M. C. , S. A. Cushman , and S. Saura . 2014. “Scale Dependence in Habitat Selection: The Case of the Endangered Brown Bear ( *Ursus arctos* ) in the Cantabrian Range (NW Spain).” International Journal of Geographical Information Science 28, no. 8: 1531–1546. 10.1080/13658816.2013.776684.

[ece374384-bib-0061] Matsuzawa, T. , T. Humle , and Y. Sugiyama , eds. 2011. The Chimpanzees of Bossou and Nimba. Vol. 395. Springer. 10.1007/978-4-431-53921-6.

[ece374384-bib-0062] Matthews, A. , and A. Matthews . 2004. “Survey of Gorillas ( *Gorilla gorilla gorilla* ) and Chimpanzees ( *Pan troglodytes troglodytes* ) in Southwestern Cameroon.” Primates 45: 15–24. 10.1007/s10329-003-0058-4. 14586801

[ece374384-bib-0063] McGarigal, K. , H. Y. Wan , K. A. Zeller , B. C. Timm , and S. A. Cushman . 2016. “Multi‐Scale Habitat Selection Modeling: A Review and Outlook.” Landscape Ecology 31: 1161–1175. 10.1007/s10980-016-0374-x.

[ece374384-bib-0065] R Core Team . 2024. “R: A Language and Environment for Statistical Computing.” R Foundation for Statistical Computing, Vienna, Austria. https://www.R‐project.org/.

[ece374384-bib-0066] Ridgeway, G. 2024. “Generalized Boosted Models: A Guide to the gbm Package.” R Package Version 2.1.9.

[ece374384-bib-0067] Robin, X. , N. Turck , A. Hainard , et al. 2023. “Package ‘pROC’.” R Package Version 1.18.5.

[ece374384-bib-0068] Sørensen, T. J. 1948. “A Method of Establishing Groups of Equal Amplitude in Plant Sociology Based on Similarity of Species Content and Its Application to Analyses of the Vegetation on Danish Commons.” Biologiske Skrifter 5: 1–34.

[ece374384-bib-0069] Stupariu, M. S. , S. A. Cushman , A. I. Pleşoianu , I. Pătru‐Stupariu , and C. Fuerst . 2022. “Machine Learning in Landscape Ecological Analysis: A Review of Recent Approaches.” Landscape Ecology 37, no. 5: 1227–1250. 10.1007/s10980-021-01366-9.

[ece374384-bib-0070] Sugiyama, Y. 1978. Bossou‐mura No Hito to Chinpanji: Nishi‐Afurika Hekichi No Seitai. Kinokuniya.

[ece374384-bib-0072] Thompson, C. M. , and K. McGarigal . 2002. “The Influence of Research Scale on Bald Eagle Habitat Selection Along the Lower Hudson River, New York (USA).” Landscape Ecology 17: 569–586. 10.1023/A:1021501231182.

[ece374384-bib-0073] Timm, B. C. , K. McGarigal , S. A. Cushman , and J. L. Ganey . 2016. “Multi‐ Scale Mexican Spotted Owl ( *Strix occidentalis lucida* ) Nest/Root Habitat Selection in Arizona and a Comparison With Single‐Scale Modeling Results.” Landscape Ecology 31: 1209–1225. 10.1007/s10980-016-0371-0.

[ece374384-bib-0074] Valavi, R. , J. Elith , J. J. Lahoz‐Monfort , and G. Guillera‐Arroita . 2018. “blockCV: An r Package for Generating Spatially or Environmentally Separated Folds for k‐Fold Cross‐Validation of Species Distribution Models.” *Biorxiv*, 357798. 10.1111/2041-210X.13107.

[ece374384-bib-0075] Valavi, R. , G. Guillera‐Arroita , J. J. Lahoz‐Monfort , and J. Elith . 2022. “Predictive Performance of Presence‐Only Species Distribution Models: A Benchmark Study With Reproducible Code.” Ecological Monographs 92, no. 1: e01486. 10.1002/ecm.1486.

[ece374384-bib-0076] Wan, H. Y. , K. McGarigal , J. L. Ganey , V. Lauret , B. C. Timm , and S. A. Cushman . 2017. “Meta‐Replication Reveals Nonstationarity in Multi‐Scale Habitat Selection of Mexican Spotted Owl.” Condor 119: 641–658. 10.1650/CONDOR-17-32.1.

[ece374384-bib-0077] Wiens, J. A. 1976. “Population Responses to Patchy Environments.” Annual Review of Ecology and Systematics 7: 81–120. 10.1146/annurev.es.07.110176.000501.

[ece374384-bib-0078] Wiens, J. A. 1989. “Spatial Scaling in Ecology.” Functional Ecology 3, no. 4: 385–397. 10.2307/2389612.

[ece374384-bib-0079] World Heritage Committee . 2023. “Mount Nimba Strict Nature Reserve.” URL Consulted on 20/12/2023. http://whc.unesco.org/en/list/155/.

[ece374384-bib-0082] Zurell, D. , J. Elith , and B. Schröder . 2012. “Predicting to New Environments: Tools for Visualizing Model Behaviour and Impacts on Mapped Distributions.” Diversity and Distributions 18, no. 6: 628–634. 10.1111/j.1472-4642.2012.00887.x.

