# Supplementary material for: Illegal Hunting and Chimpanzee Distribution in the Seringbara Region of the Mount Nimba Strict Nature Reserve: A Multi‐Scale Modelling Approach
Source: Ecol Evol. 2026 Oct 5;16(10):e74384. doi: 10.1002/ece3.74384 (PMC13638268; doi:10.1002/ece3.74384)
Supplement: Supplementary file 1 [file ECE3-16-e74384-s001.docx]

# SUPPLEMENTARY MATERIALS

## Supplementary Code

All R scripts used in this study are available on the following Anonymous GitHub repository: <https://anonymous.4open.science/r/Seringbara-8E36>

## Supplementary Tables

**Table S1. List of environmental variables selected for modelling purposes.**

| **Predictor variable** | **Description** | **Source** | **Resolution** | **Reference** |
| --- | --- | --- | --- | --- |
| Elevation | Height above sea level | Multi-Error-Removed Improved-Terrain | 90 m | Yamazaki, 2018 |
| Tree cover | Percentage of ground covered by woody vegetation greater than 5m in height | Global Land Analysis and Discovery | 30 m | Hansen *et al.*, 2013 |
| Continuous heat-insolation load index (CHILI) | Measurement of heat load considering steepness of slope and aspect | Shuttle Radar Topography Mission | 30 m | Theobald *et al.*, 2015  - [Conservation Science Partners](https://csp-inc.org/) |
| Enhanced vegetation index (EVI) | Index of relative biomass | Landsat 8 | 30 m | Huete *et al.*, 2002 |
| Greenness* | Measure of photosynthetically active vegetation | Landsat 8 | 30 m | Baig *et al.*, 2014 |
| Wetness | Soil moisture content | Landsat 8 | 30 m | Baig *et al.*, 2014 |
| Brightness* | Soil brightness | Landsat 8 | 30 m | Baig *et al.*, 2014 |
| Slope | Steepness of a surface | Geomorpho90 | 90 m | Amatulli *et al.*, 2020 |
| Aspect | Direction a slope faces | Geomorpho90 | 90 m | Amatulli *et al.*, 2020 |
| Compound topographic index (CTI) | Steady-state wetness index | Geomorpho90 | 90 m | Amatulli *et al.*, 2020 |
| Roughness* | Measure of surface roughness | Geomorpho90 | 90 m | Amatulli *et al.*, 2020 |
| Topographic position index (TPI) | Difference between elevation at one point and the mean elevation around it | Geomorpho90 | 90 m | Amatulli *et al*., 2020 |
| Distance to village | Euclidean distance between a cell and nearest village | OpenStreetMap | Vector | OpenStreetMap contributors, 2015 |
| Distance to road | Euclidean distance between a cell and nearest road | OpenStreetMap | Vector | OpenStreetMap contributors, 2015 |
| Distance to river | Euclidean distance between a cell and nearest permanent river or stream | WSP - Nimba Mountains - Key Watercourses | Vector | WSP Global Inc., 2020 |
| Distance to research camp | Euclidean distance between a cell and nearest camp | Koops & Fitzgerald | Vector | Field data |

After performing a correlation analysis, this initial set of 16 variables was reduced to 13 for use in the final models (variables excluded are marked with *).

**Table S2. Optimal scales for variables in the chimpanzee feeding models, determined by mean cross-validated area under the receiver operating characteristic curve (cv AUC) for 100 independent analyses and 100 sets of pseudo-absence points using univariate GLM and BRT modelling.**

| Predictor variable | Modelling technique | Scale selected | cross-validated AUC |
| --- | --- | --- | --- |
| Aspect | GLM | 2,000 m | 0.611 |
| Aspect | BRT | 1,500 m | 0.579 |
| Brightness | GLM | 30 m | 0.537 |
| Brightness | BRT | 500 m | 0.613 |
| CTI | GLM | 1,000 m | 0.651 |
| CTI | BRT | 1,500 m | 0.625 |
| Elevation | GLM | 250 m | 0.644 |
| Elevation | BRT | 30 m | 0.642 |
| EVI | GLM | 1,500 m | 0.584 |
| EVI | BRT | 100 m | 0.593 |
| Greenness | GLM | 1,500 m | 0.585 |
| Greenness | BRT | 1,000 m | 0.567 |
| CHILI | GLM | 1,000 m | 0.594 |
| CHILI | BRT | 2,000 m | 0.607 |
| Roughness | GLM | 250 m | 0.656 |
| Roughness | BRT | 250 m | 0.629 |
| Slope | GLM | 250 m | 0.655 |
| Slope | BRT | 250 m | 0.631 |
| TPI | GLM | 2,000 m | 0.643 |
| TPI | BRT | 2,000 m | 0.583 |
| Tree cover | GLM | 1,500 m | 0.579 |
| Tree cover | BRT | 2,000 m | 0.570 |
| Wetness | GLM | 1,500 m | 0.619 |
| Wetness | BRT | 1,500 m | 0.608 |

**Table S3. Optimal scales for variables in the chimpanzee nesting model, determined by mean cross-validated area under the receiver operating characteristic curve (cv AUC) from 100 runs and 100 pseudo-absence sets using univariate GLM and BRT modelling.**

| Predictor variable | Modelling technique | Scale selected | cross-validated AUC |
| --- | --- | --- | --- |
| Aspect | GLM | 1,500 m | 0.612 |
| Aspect | BRT | 1,500 m | 0.585 |
| Brightness | GLM | 30 m | 0.551 |
| Brightness | BRT | 500 m | 0.614 |
| CTI | GLM | 1,500 m | 0.656 |
| CTI | BRT | 500 m | 0.658 |
| Elevation | GLM | 250 m | 0.633 |
| Elevation | BRT | 1,000 m | 0.614 |
| EVI | GLM | 1,500 m | 0.584 |
| EVI | BRT | 100 m | 0.618 |
| Greenness | GLM | 1,500 m | 0.585 |
| Greenness | BRT | 1,000 m | 0.567 |
| CHILI | GLM | 100 m | 0.624 |
| CHILI | BRT | 1,500 m | 0.615 |
| Roughness | GLM | 250 m | 0.721 |
| Roughness | BRT | 250 m | 0.683 |
| Slope | GLM | 250 m | 0.723 |
| Slope | BRT | 500 m | 0.683 |
| TPI | GLM | 2,000 m | 0.661 |
| TPI | BRT | 2,000 m | 0.631 |
| Tree cover | GLM | 1,000 m | 0.594 |
| Tree cover | BRT | 2,000 m | 0.582 |
| Wetness | GLM | 1,500 m | 0.633 |
| Wetness | BRT | 2,000 m | 0.646 |

**Table S4. Optimal scales for variables in the human hunting model, determined by mean cross-validated area under the receiver operating characteristic curve (cv AUC) from 100 runs and 100 pseudo-absence sets using univariate GLM and BRT modelling.**

| Predictor variable | Modelling technique | Scale selected | cross-validated AUC |
| --- | --- | --- | --- |
| Aspect | GLM | 1,000 m | 0.648 |
| Aspect | BRT | 2,000 m | 0.625 |
| Brightness | GLM | 2,000 m | 0.631 |
| Brightness | BRT | 250 m | 0.610 |
| CTI | GLM | 500 m | 0.707 |
| CTI | BRT | 1,000 m | 0.636 |
| Elevation | GLM | 30 m | 0.698 |
| Elevation | BRT | 30 m | 0.616 |
| EVI | GLM | 1,500 m | 0.645 |
| EVI | BRT | 1,500 m | 0.597 |
| Greenness | GLM | 1,500 m | 0.632 |
| Greenness | BRT | 1,000 m | 0.603 |
| CHILI | GLM | 1,500 m | 0.594 |
| CHILI | BRT | 2,000 m | 0.624 |
| Roughness | GLM | 1,500 m | 0.641 |
| Roughness | BRT | 100 m | 0.604 |
| Slope | GLM | 1,000 m | 0.638 |
| Slope | BRT | 100 m | 0.592 |
| TPI | GLM | 2,000 m | 0.689 |
| TPI | BRT | 1,000 m | 0.618 |
| Tree cover | GLM | 1,000 m | 0.742 |
| Tree cover | BRT | 500 m | 0.703 |
| Wetness | GLM | 1,500 m | 0.742 |
| Wetness | BRT | 1,500 m | 0.652 |

**Table S5. Predictor variables ranked by effect size ß (standardised regression coefficients) per averaged binomial model trained with the GLM approach.**

| **feed_30m** | **feed_scale** | **nest_30m** | **nest_scale** | **hunt_30m** | **hunt_scale** |
| --- | --- | --- | --- | --- | --- |
| Distance to camp (ß = 9.56E-04)^*1^ | Slope (250 m) (ß = 0.105)^*8^ | Distance to camp (ß = 0.00111)^*13^ | Slope (250 m) (ß = 0.193)^*18^ | Distance to camp (ß = 0.00169)^*20^ | Distance to camp (ß = 0.00154)^*22^ |
| Slope (ß = 0.0715)^*2^ | Distance to camp (ß = 6.49E-04)^*9^ | Slope (ß = 0.139)^*14^ | Distance to camp (ß = 9.38E-04)^*19^ | Distance to road (ß = -9.82E-04)^*21^ | CTI (500 m) (ß = -0.962)^*23^ |
| CTI (ß = -0.286)^*3^ | Aspect (2000 m) (ß = 0.0319)^*10^ | EVI (ß = 7.59)^*15^ | — | — | Tree cover (1000 m) (ß = -0.214)^*24^ |
| Distance to village (ß = -1.92E-04)^*4^ | Distance to river (ß = -4.04E-04)^*11^ | Aspect (ß = -0.00351)^*16^ | — | — | — |
| EVI (ß = 4.52)^*5^ | TPI (2000 m) (ß = -1.09)^*12^ | Distance to village (ß = -2.60E-04)^*17^ | — | — | — |
| TPI (ß = -0.0369)^*6^ | — | — | — | — | — |
| Aspect (ß = -0.00188)^*7^ | — | — | — | — | — |

**Table S6. Top five predictor variables ranked by relative importance (in percentage) per kind of model trained with the BRT approach.**

| **feed_30m** | **feed_scale** | **nest_30m** | **nest_scale** | **hunt_30m** | **hunt_scale** |
| --- | --- | --- | --- | --- | --- |
| Elevation (35.3%) | Elevation at 30 m (27.2%) | Elevation (27.7%) | Elevation at 100 m (20.9%) | Distance to camp (34.9%) | Tree cover at 500 m (19.5%) |
| Distance to camp (15.6%) | TPI at 2,000 m (11.9`%) | Distance to camp (15.1%) | Slope at 500 m (18.2%) | Distance to river (9.9%) | Distance to camp (18.7%) |
| Distance to road (10.8%) | EVI at 100 m (8.8%) | Slope (13.8%) | Wetness at 2,000 m (11.3%) | Elevation (8%) | Wetness at 1,500 m (14.5%) |
| Distance to village (7.6%) | Distance to camp (8.4%) | EVI (9.9%) | TPI at 2,000 m (9.8%) | CTI (7.9%) | Distance to river (9%) |
| Slope (7%) | Slope at 250 m (8.3%) | CTI (7.1%) | CTI at 500 m (8.9%). | Distance to road (5.9%) | Aspect at 2,000 m (8%). |

**Table S7. Summary of 100 averaged binomial GLM runs using the dredge function from the MuMIn package for the chimpanzee feeding model with single-scale variables.**

| Variable | Estimate | Std. Error | Adjusted SE | 95% CI lower | 95% CI upper | Importance |
| --- | --- | --- | --- | --- | --- | --- |
| (Intercept) | -4.052 | 1.884 | 1.886 | — | — | — |
| Aspect | -1.88E-03 | 9.27E-04 | 9.29E-04 | -0.0037 | -6.31E-05 | 0.6959 |
| CTI | -0.286 | 0.091 | 0.091 | -0.4644 | -0.1078 | 0.9274 |
| Distance to camp | 9.56E-04 | 1.47E-04 | 1.47E-04 | 6.67E-04 | 0.0012 | 0.9997 |
| Distance to road | -2.39E-04 | 1.46E-04 | 1.46E-04 | -5.25E-04 | 4.75E-05 | 0.5908 |
| Distance to village | -1.92E-04 | 7.76E-05 | 7.77E-05 | -3.45E-04 | -4.01E-05 | 0.7744 |
| EVI | 4.522 | 2.013 | 2.016 | 0.5713 | 8.4729 | 0.7705 |
| Slope | 0.072 | 0.022 | 0.022 | 0.0293 | 0.1137 | 0.9443 |
| Tree cover | 0.011 | 0.012 | 0.012 | -0.0120 | 0.0337 | 0.4375 |
| CHILI | 2.16E-03 | 0.012 | 0.012 | -0.0213 | 0.0256 | 0.3573 |
| TPI | -0.037 | 0.017 | 0.017 | -0.0705 | -0.0033 | 0.7318 |
| Distancet to river | -1.32E-04 | 1.43E-04 | 1.44E-04 | -4.13E-04 | 1.50E-04 | 0.3924 |
| Elevation | 5.82E-04 | 9.91E-04 | 9.92E-04 | -0.0014 | 0.0025 | 0.3960 |
| Wetness | -3.358 | 15.516 | 15.539 | -33.8140 | 27.0978 | 0.3247 |

**Table S8. Summary of 100 averaged binomial GLM runs using the dredge function from the MuMIn package for the chimpanzee feeding model with multi-scale variables.**

| Variable | Estimate | Std. Error | Adjusted SE | 95% CI lower | 95% CI upper | Importance |
| --- | --- | --- | --- | --- | --- | --- |
| (Intercept) | -3.300 | 6.750 | 6.755 | N/A | N/A | N/A |
| Aspect (2000 m) | 0.032 | 0.011 | 0.011 | 0.0100 | 0.0538 | 0.7810 |
| CTI (1000 m) | -0.666 | 0.402 | 0.402 | -1.4544 | 0.1217 | 0.5875 |
| Distance to camp | 6.49E-04 | 2.17E-04 | 2.17E-04 | 2.24E-04 | 0.0011 | 0.8802 |
| Distance to river | -4.04E-04 | 1.89E-04 | 1.89E-04 | -7.75E-04 | -3.34E-05 | 0.7018 |
| Slope (250 m) | 0.105 | 0.028 | 0.028 | 0.0491 | 0.1608 | 0.9476 |
| TPI (2000 m) | -1.094 | 0.544 | 0.544 | -2.1606 | -0.0265 | 0.6221 |
| Tree cover (1500 m) | -0.064 | 0.034 | 0.034 | -0.1309 | 0.0030 | 0.5862 |
| Wetness (1500 m) | 42.056 | 36.988 | 37.028 | -30.5163 | 114.6293 | 0.4829 |
| Distance to road | -1.46E-04 | 2.14E-04 | 2.14E-04 | -5.66E-04 | 2.74E-04 | 0.4566 |
| Distance to village | -6.29E-05 | 1.87E-04 | 1.88E-04 | -4.31E-04 | 3.05E-04 | 0.5020 |
| EVI (1500 m) | 11.056 | 8.035 | 8.044 | -4.7093 | 26.8211 | 0.4702 |
| Elevation (250 m) | 2.43E-03 | 1.36E-03 | 1.36E-03 | -2.33E-04 | 0.0051 | 0.6079 |
| CHILI (1000 m) | -0.042 | 0.036 | 0.036 | -0.1127 | 0.0292 | 0.4546 |

**Table S9. Summary of 100 averaged binomial GLM runs using the dredge function from the MuMIn package for the chimpanzee nesting model with single-scale variables.**

| Variable | Estimate | Std. Error | Adjusted SE | 95% CI lower | 95% CI upper | Importance |
| --- | --- | --- | --- | --- | --- | --- |
| (Intercept) | -2.709 | 3.552 | 3.562 | N/A | N/A | N/A |
| Aspect | -3.51E-03 | 1.59E-03 | 1.60E-03 | -0.0066 | -3.76E-04 | 0.7560 |
| Distance to camp | 1.11E-03 | 2.50E-04 | 2.51E-04 | 6.22E-04 | 0.0016 | 0.9942 |
| Distance to village | -2.60E-04 | 1.22E-04 | 1.23E-04 | -5.01E-04 | -1.91E-05 | 0.6804 |
| EVI | 7.586 | 3.409 | 3.423 | 0.8777 | 14.2946 | 0.7564 |
| CHILI | -0.021 | 0.019 | 0.019 | -0.0587 | 0.0168 | 0.4264 |
| Slope | 0.139 | 0.036 | 0.036 | 0.0680 | 0.2093 | 0.9492 |
| CTI | -0.071 | 0.124 | 0.125 | -0.3153 | 0.1739 | 0.3614 |
| Distance to road | -2.36E-04 | 2.34E-04 | 2.35E-04 | -6.96E-04 | 2.24E-04 | 0.4220 |
| Tree cover | 7.94E-03 | 0.025 | 0.025 | -0.0405 | 0.0564 | 0.3448 |
| TPI | 0.022 | 0.022 | 0.022 | -0.0204 | 0.0643 | 0.4101 |
| Elevation | -4.02E-04 | 1.48E-03 | 1.49E-03 | -0.0033 | 0.0025 | 0.3474 |
| Distance to river | -1.16E-04 | 2.53E-04 | 2.54E-04 | -6.13E-04 | 3.82E-04 | 0.3402 |
| Wetness | 0.890 | 29.628 | 29.751 | -57.4210 | 59.2014 | 0.3350 |

**Table S10. Summary of 100 averaged binomial GLM runs using the dredge function from the MuMIn package for the chimpanzee nesting model with multi-scale variables.**

| Variable | Estimate | Std. Error | Adjusted SE | 95% CI lower | 95% CI upper | Importance |
| --- | --- | --- | --- | --- | --- | --- |
| (Intercept) | -4.825 | 6.845 | 6.857 | N/A | N/A | N/A |
| Aspect (1500 m) | 0.017 | 0.010 | 0.011 | -0.0034 | 0.0378 | 0.5430 |
| Distance to camp | 9.38E-04 | 3.21E-04 | 3.22E-04 | 3.06E-04 | 0.0016 | 0.8473 |
| EVI (1500 m) | 13.446 | 10.467 | 10.504 | -7.1413 | 34.0336 | 0.4891 |
| CHILI (100 m) | -0.016 | 0.019 | 0.019 | -0.0531 | 0.0213 | 0.3626 |
| Slope (250 m) | 0.193 | 0.043 | 0.043 | 0.1082 | 0.2778 | 0.9837 |
| Wetness (1500 m) | 16.994 | 43.192 | 43.326 | -67.9238 | 101.9119 | 0.4109 |
| Tree cover (1000 m) | -0.059 | 0.044 | 0.045 | -0.1469 | 0.0280 | 0.4754 |
| Distance to road | -1.03E-04 | 3.06E-04 | 3.07E-04 | -7.05E-04 | 4.99E-04 | 0.4264 |
| TPI (2000 m) | -0.584 | 0.820 | 0.823 | -2.1979 | 1.0295 | 0.3844 |
| Distance to river | -4.36E-04 | 3.03E-04 | 3.05E-04 | -0.0010 | 1.61E-04 | 0.4933 |
| Distance to village | -2.74E-04 | 2.00E-04 | 2.00E-04 | -6.66E-04 | 1.19E-04 | 0.5023 |
| Elevation (250 m) | 1.66E-03 | 1.73E-03 | 1.73E-03 | -0.0017 | 0.0051 | 0.4030 |
| CTI (1000 m) | -0.281 | 0.603 | 0.605 | -1.4675 | 0.9050 | 0.3414 |

**Table S11. Summary of 100 averaged binomial GLM runs using the dredge function from the MuMIn package for the human hunting model with single-scale variables.**

| Variable | Estimate | Std. Error | Adjusted SE | 95% CI lower | 95% CI upper | Importance |
| --- | --- | --- | --- | --- | --- | --- |
| (Intercept) | -2.617 | 5.311 | 5.341 | N/A | N/A | N/A |
| Distance to camp | 1.69E-03 | 4.25E-04 | 4.29E-04 | 8.47E-04 | 0.0025 | 0.9807 |
| Distance to village | 2.20E-04 | 2.52E-04 | 2.55E-04 | -2.79E-04 | 7.19E-04 | 0.4130 |
| Tree cover | -0.047 | 0.047 | 0.047 | -0.1394 | 0.0446 | 0.3922 |
| Wetness | 62.826 | 46.951 | 47.375 | -30.0277 | 155.6802 | 0.4696 |
| Distance to river | 6.76E-04 | 4.17E-04 | 4.20E-04 | -1.48E-04 | 0.0015 | 0.5030 |
| Distance to road | -9.82E-04 | 4.85E-04 | 4.89E-04 | -0.0019 | -2.34E-05 | 0.6458 |
| TPI | -3.22E-03 | 0.035 | 0.035 | -0.0720 | 0.0656 | 0.3397 |
| Aspect | 2.37E-03 | 2.63E-03 | 2.66E-03 | -0.0028 | 0.0076 | 0.3501 |
| EVI | 5.722 | 5.665 | 5.716 | -5.4812 | 16.9247 | 0.4103 |
| Slope | 0.038 | 0.051 | 0.052 | -0.0635 | 0.1399 | 0.3551 |
| Elevation | 2.25E-03 | 2.43E-03 | 2.45E-03 | -0.0026 | 0.0070 | 0.4331 |
| CTI | -0.229 | 0.171 | 0.172 | -0.5666 | 0.1086 | 0.4571 |
| CHILI | 0.016 | 0.027 | 0.027 | -0.0379 | 0.0692 | 0.3421 |

**Table S12. Summary of 100 averaged binomial GLM runs using the dredge function from the MuMIn package for the human hunting model with multi-scale variables.**

| Variable | Estimate | Std. Error | Adjusted SE | 95% CI lower | 95% CI upper | Importance |
| --- | --- | --- | --- | --- | --- | --- |
| (Intercept) | -5.661 | 15.700 | 15.756 | N/A | N/A | N/A |
| CTI (500 m) | -0.962 | 0.432 | 0.435 | -1.8147 | -0.1092 | 0.7048 |
| Distance to camp | 1.54E-03 | 5.28E-04 | 5.32E-04 | 4.95E-04 | 0.0026 | 0.8919 |
| Distance to river | 9.29E-04 | 5.55E-04 | 5.60E-04 | -1.69E-04 | 0.0020 | 0.5486 |
| Slope (1000 m) | -0.123 | 0.104 | 0.104 | -0.3279 | 0.0812 | 0.4247 |
| Tree cover (1000 m) | -0.214 | 0.090 | 0.090 | -0.3911 | -0.0365 | 0.7017 |
| Aspect (1000 m) | 0.024 | 0.015 | 0.015 | -0.0058 | 0.0540 | 0.5025 |
| EVI (1500 m) | 41.100 | 23.135 | 23.296 | -4.5589 | 86.7593 | 0.5306 |
| Wetness (1500 m) | -115.209 | 91.802 | 92.467 | -296.4400 | 66.0224 | 0.5280 |
| Distance to road | 3.29E-04 | 6.42E-04 | 6.48E-04 | -9.40E-04 | 0.0016 | 0.3809 |
| TPI (2000 m) | -2.569 | 1.416 | 1.427 | -5.3665 | 0.2283 | 0.5318 |
| Elevation (30 m) | 6.29E-04 | 3.49E-03 | 3.52E-03 | -0.0063 | 0.0075 | 0.4717 |
| Distance to village | -2.08E-04 | 3.63E-04 | 3.66E-04 | -9.25E-04 | 5.08E-04 | 0.3877 |

**Table S13. Spatial correlation coefficients and averaged absolute differences between predicted chimpanzee activity (Model 1) and human hunting activity (Model 2) surfaces across modelling approaches (BRT, GLM) and scale methods (single-scale vs. multi-scale).**

| Model type | Model 1 | Model 2 | Correlation | Absolute Difference |
| --- | --- | --- | --- | --- |
| BRT | feed_30m | hunt_30m | 0.427 | 0.152 |
| BRT | feed_scale | hunt_scale | 0.534 | 0.167 |
| BRT | nest_30m | hunt_30m | 0.437 | 0.170 |
| BRT | nest_scale | hunt_scale | 0.383 | 0.213 |
| GLM | feed_30m | hunt_30m | 0.697 | 0.142 |
| GLM | feed_scale | hunt_scale | 0.423 | 0.264 |
| GLM | nest_30m | hunt_30m | 0.650 | 0.160 |
| GLM | nest_scale | hunt_scale | 0.605 | 0.195 |

## Supplementary Figures

**Figure S1a. Response curves of the 100 BRT models for the feeding model using single-scale variables.** The five most important predictor variables, ranked by percent contribution, are elevation (35.3%), distance to camp (15.6%), distance to road (10.8%), distance to village (7.6%), and slope (7%).

**Figure S1b. Predicted ecological suitability map (left) and standard deviation map (right), based on the average of the 100 BRT models for the feeding model using single-scale variables.** Dark colours indicate higher values—dark green represents high ecological suitability on the left map, while dark red represents high standard deviation on the right map.

**Figure S2a. Response curves of the 100 BRT models for the feeding model using scale-optimised variables.** The five most important predictor variables, ranked by percent contribution, are elevation at 30 m (27.2%), TPI at 2000 m (11.9%), EVI at 100 m (8.8%), distance to camp (8.4%) and slope at 250 m (8.3%).

**Figure S2b. Predicted ecological suitability map (left) and standard deviation map (right), based on the average of the 100 BRT models for the feeding model using scale-optimised variables.** Dark colours indicate higher values—dark green represents high ecological suitability on the left map, while dark red represents high standard deviation on the right map.

**Figure S3a. Response curves of the 100 BRT models for the nesting model using single-scale variables.** The five most important predictor variables, ranked by percent contribution, are elevation (27.7%), distance to camp (15.1%), slope (13.8%), EVI (9.9%), and CTI (7.1%).

**Figure S3b. Predicted ecological suitability map (left) and standard deviation map (right), based on the average of the 100 BRT models for the nesting model using single-scale variables.** Dark colours indicate higher values—dark green represents high ecological suitability on the left map, while dark red represents high standard deviation on the right map.

**Figure S4a. Response curves of the 100 BRT models for the nesting model using scale-optimised variables.** The five most important predictor variables, ranked by percent contribution, are elevation at 100 m (20.9%), slope at 500 m (18.2%), wetness at 2000 m (11.3%), TPI at 2000 m (9.8%), and CTI at 500 m (8.9%).

**Figure S4b. Predicted ecological suitability map (left) and standard deviation map (right), based on the average of the 100 BRT models for the nesting model using scale-optimised variables.** Dark colours indicate higher values—dark green represents high ecological suitability on the left map, while dark red represents high standard deviation on the right map.

**Figure S5a. Response curves of the 100 BRT models for the hunting model using single-scale variables.** The five most important predictor variables, ranked by percent contribution, are distance to camp (34.9%), distance to river (9.9%), elevation (8%), CTI (7.9%) and distance to road (5.9%).

**Figure S5b. Predicted ecological suitability map (left) and standard deviation map (right), based on the average of the 100 BRT models for the hunting model using single-scale variables.** Dark colours indicate higher values—dark green represents high ecological suitability on the left map, while dark red represents high standard deviation on the right map.

**Figure S6a. Response curves of the 100 BRT models for the hunting model using scale-optimised variables.** The five most important predictor variables, ranked by percent contribution, are tree cover at 500 m (19.5%), distance to camp (18.7%), wetness at 1500 m (14.5%), distance to river (9%) and aspect at 2000 m (8%).

**Figure S6b. Predicted ecological suitability map (left) and standard deviation map (right), based on the average of the 100 BRT models for the hunting model using scale-optimised variables.** Dark colours indicate higher values—dark green represents high ecological suitability on the left map, while dark red represents high standard deviation on the right map.

**Figure S7a. Box plot of the predictor variables, ranked by percent contribution, for the chimpanzee feeding model using single-scale variables (A), using scale-optimised variables (B), and for the human hunting model using single-scale variables (C).**

**Figure S7b. Box plot of the predictor variables, ranked by percent contribution, for the human hunting model using scale-optimised variables (D), for the chimpanzee nesting model using single-scale variables (E) and using scale-optimised variables (F).**

**Figure S8. Plots of the correlation matrix of the different environmental variables that showed high correlation. Correlation coefficients were calculated using Spearman’s correlation.** Dark colours represent a high correlation coefficient, with blue indicating a positive correlation and red indicating a negative correlation. **A.** Single-scale chimpanzee feeding/travelling model **B.** Single-scale human hunting model **C.** Single-scale chimpanzee nesting model **D.** Multi-scale human hunting model.

**Figure S9. Predicted ecological suitability map (left) and standard deviation map (right), based on the 100 averaged GLM for the chimpanzee feeding model using single-scale variables.** Dark colours indicate higher values—dark green represents high ecological suitability on the left map, while dark red represents high standard deviation on the right map.

**Figure S10. Predicted ecological suitability map (left) and standard deviation map (right), based on the 100 averaged GLM for the chimpanzee feeding model using scale-optimised variables.** Dark colours indicate higher values—dark green represents high ecological suitability on the left map, while dark red represents high standard deviation on the right map.

**Figure S11. Predicted ecological suitability map (left) and standard deviation map (right), based on the 100 averaged GLM for the human hunting model using single-scale variables.** Dark colours indicate higher values—dark green represents high ecological suitability on the left map, while dark red represents high standard deviation on the right map.

**Figure S12. Predicted ecological suitability map (left) and standard deviation map (right), based on the 100 averaged GLM for the human hunting model using scale-optimised variables.** Dark colours indicate higher values—dark green represents high ecological suitability on the left map, while dark red represents high standard deviation on the right map.

**Figure S13. Predicted ecological suitability map (left) and standard deviation map (right), based on the 100 averaged GLM for the human hunting model using single-scale variables.** Dark colours indicate higher values—dark green represents high ecological suitability on the left map, while dark red represents high standard deviation on the right map.

**Figure S14. Predicted ecological suitability map (left) and standard deviation map (right), based on the 100 averaged GLM for the human hunting model using scale-optimised variables.** Dark colours indicate higher values—dark green represents high ecological suitability on the left map, while dark red represents high standard deviation on the right map.

**Figure S15. Response curves for the five most influential predictor variables, ranked by their relative importance (in percentage) in 100 replicate BRT models trained for each activity type.** The curves are shown for both single-scale and multi-scale variables.

**Figure S16. Map illustrating the differences in ecological suitability between the BRT chimpanzee feeding/travelling model and the BRT human hunting model using single-scale variables (left) and multi-scale variables (right).** Low values, i.e. darker reds, indicate reduced ecological suitability for chimpanzee feeding/travelling and/or increased ecological suitability for human hunting, while high values, i.e. darker blues, indicate the opposite.

**Figure S17. Map illustrating the differences in ecological suitability between the BRT chimpanzee nesting model and the BRT human hunting model using single-scale variables (left) and multi-scale variables (right).** Low values, i.e. darker reds, indicate reduced ecological suitability for chimpanzee nesting and/or increased ecological suitability for human hunting, while high values, i.e. darker blues, indicate the opposite.

**Figure S18. Map illustrating the differences in ecological suitability between the GLM chimpanzee feeding/travelling model and the GLM human hunting model using single-scale variables (left) and multi-scale variables (right).** Low values, i.e. darker reds, indicate reduced ecological suitability for chimpanzee feeding/travelling and/or increased ecological suitability for human hunting, while high values, i.e. darker blues, indicate the opposite.

**Figure S19. Map illustrating the differences in ecological suitability between the GLM chimpanzee nesting model and the GLM human hunting model using single-scale variables (left) and multi-scale variables (right).** Low values, i.e. darker reds, indicate reduced ecological suitability for chimpanzee nesting and/or increased ecological suitability for human hunting, while high values, i.e. darker blues, indicate the opposite.

**Figure S20. Map showing areas of overlap between the BRT chimpanzee feeding/travelling model and the BRT human hunting model using single-scale variables (left) and multi-scale variables (right).** Higher values, i.e. darker reds, indicate an increased probability of interaction between hunting activities and chimpanzee feeding/travelling activities.

**Figure S21. Map showing areas of overlap between the BRT chimpanzee nesting model and the BRT human hunting model using single-scale variables (left) and multi-scale variables (right).** Higher values, i.e. darker reds, indicate an increased probability of interaction between hunting activities and chimpanzee nesting sites.

**Figure S22. Map showing areas of overlap between the GLM chimpanzee feeding/travelling model and the GLM human hunting model using single-scale variables (left) and multi-scale variables (right).** Higher values, i.e. darker reds, indicate an increased probability of interaction between hunting activities and chimpanzee feeding/travelling activities.

**Figure S23. Map showing areas of overlap between the GLM chimpanzee nesting model and the GLM human hunting model using single-scale variables (left) and multi-scale variables (right).** Higher values, i.e. darker reds, indicate an increased probability of interaction between hunting activities and chimpanzee nesting sites.
